# Non-SMC Element 2 (NSMCE2) of the SMC5/6 Complex Helps to Resolve Topological Stress

**DOI:** 10.3390/ijms17111782

**Published:** 2016-10-26

**Authors:** Dideke E. Verver, Yi Zheng, Dave Speijer, Ron Hoebe, Henk L. Dekker, Sjoerd Repping, Jan Stap, Geert Hamer

**Affiliations:** 1 Center for Reproductive Medicine, Amsterdam Research Institute Reproduction and Development, Academic Medical Center, University of Amsterdam, 1105 AZ Amsterdam, The Netherlands; emma.verver@nikon.com (D.E.V.); y.zheng@amc.uva.nl (Y.Z.); s.repping@amc.uva.nl (S.R.); 2 Department of Medical Biochemistry, Academic Medical Center, University of Amsterdam, 1105 AZ Amsterdam, The Netherlands; d.speijer@amc.uva.nl; 3 Department of Cell Biology and Histology, Academic Medical Center, University of Amsterdam, 1105 AZ Amsterdam, The Netherlands; r.a.hoebe@amc.uva.nl (R.H.); j.stap@amc.uva.nl (J.S.); 4 Mass Spectrometry of Biomacromolecules, Swammerdam Institute for Life Sciences, University of Amsterdam, 1090 GE Amsterdam, The Netherlands; h.l.dekker@uva.nl

**Keywords:** Structural Maintenance of Chromosomes 5/6 complex (SMC5/6), Non-SMC Element 2 (NSMCE2), Topoisomerase II α (TOP2A), DNA double-strand breaks (DSBs), Ionizing Radiation (IR), CRISPR-Cas9

## Abstract

The structural maintenance of chromosomes (SMC) protein complexes shape and regulate the structure and dynamics of chromatin, thereby controlling many chromosome-based processes such as cell cycle progression, differentiation, gene transcription and DNA repair. The SMC5/6 complex is previously described to promote DNA double-strand breaks (DSBs) repair by sister chromatid recombination, and found to be essential for resolving recombination intermediates during meiotic recombination. Moreover, in budding yeast, SMC5/6 provides structural organization and topological stress relief during replication in mitotically dividing cells. Despite the essential nature of the SMC5/6 complex, the versatile mechanisms by which SMC5/6 functions and its molecular regulation in mammalian cells remain poorly understood. By using a human osteosarcoma cell line (U2OS), we show that after the CRISPR-Cas9-mediated removal of the SMC5/6 subunit NSMCE2, treatment with the topoisomerase II inhibitor etoposide triggered an increased sensitivity in cells lacking NSMCE2. In contrast, NSMCE2 appeared not essential for a proper DNA damage response or cell survival after DSB induction by ionizing irradiation (IR). Interestingly, by way of immunoprecipitations (IPs) and mass spectrometry, we found that the SMC5/6 complex physically interacts with the DNA topoisomerase II α (TOP2A). We therefore propose that the SMC5/6 complex functions in resolving TOP2A-mediated DSB-repair intermediates generated during replication.

## 1. Introduction

The structural maintenance of chromosome (SMC) protein complexes shape and determine chromatin structure and function and are therefore implicated in many, if not all, fundamental chromosome-based processes. The three SMC protein complexes, cohesin, condensin and SMC5/6, all consist of two SMC subunits and several non-SMC elements, the NSMCEs. The resulting ring-like complexes possess the capacity to hold two DNA double-strands together, and are therefore able to physically shape the DNA in specific chromatin structures [1,2]. By doing so, SMC complexes control chromosome segregation, DNA repair, transcription and replication, among other processes [1,3,4,5]. Of the three SMC complexes, the SMC5/6 complex has been most directly and exclusively described to be involved in DNA damage repair and genomic integrity maintenance [6,7,8].

In mammals, SMC6 is highly expressed in the testis [9,10] and we have recently found SMC6 to be involved in crucial processes during mouse and human spermatogenesis, including spermatogonial differentiation and meiosis [9,10]. In various organisms, ranging from yeast to humans, SMC5/6 is involved in numerous meiotic processes [11] such as chromosome segregation [9,10,12,13], homologous chromosome synapsis [9,12,13,14,15,16] and meiotic sex chromosome inactivation [10,13]. Co-localization studies have suggested that SMC5/6 prevents dangerous and error-prone homologous recombination (HR) in highly repetitive, densely packed DNA regions such as the rDNA and pericentromeric heterochromatin [10,13,15,16,17,18]. SMC5/6 seems to be involved in double-strand break (DSB) repair as it is enriched at DSB sites in budding yeast and *C. elegans* [12,14,16]; it localizes side by side with RAD51 in budding yeast and humans [9,12,16] and its deletion results in an increase in RAD51 foci and chromosome fragmentation in *C. elegans* [14]. Furthermore, Smc5/6 has been found to play a role in the resolution of meiotic recombination intermediates and mutations of Smc5, Smc6 or the SUMO ligase domain of Nse2 lead to the accumulation of toxic joint molecules in yeast and *C. elegans* [12,15,16,19,20,21,22].

In budding and fission yeast the Smc5/6 complex is essential for the maintenance of replication fork stability, the prevention of joint molecules and the resolution of such joint molecules that would otherwise lead to mitotic failure (reviewed in [23,24,25]). In mice, ablation of *SMC6* results in embryonic lethality, whilst a mutation in its ATP hydrolysis motif only generates a mild phenotype [26]. NSMCE2 has also been shown to be essential for mouse development and it can suppress cancer and aging by limiting recombination and facilitating chromosome segregation [27]. In line with these studies, a recent paper describes that depletion of *SMC5* in mouse embryonic stem cells led to accumulation of cells in G2 and subsequent mitotic failure and apoptosis [28].

From this increasing amount of data, it has become overwhelmingly clear that SMC5/6 is essential for maintaining genomic integrity by a variety of means. However, the exact roles of the SMC5/6 complex in mammalian especially human cells remain poorly understood. By using a commonly used human osteosarcoma cell line (U2OS), we extended our knowledge regarding the roles of SMC5/6 in human genome integrity maintenance.

## 2. Results

### 2.1. CRISPR-Cas9-Mediated Targeting of the SMC5/6 Complex

**Figure 1:**
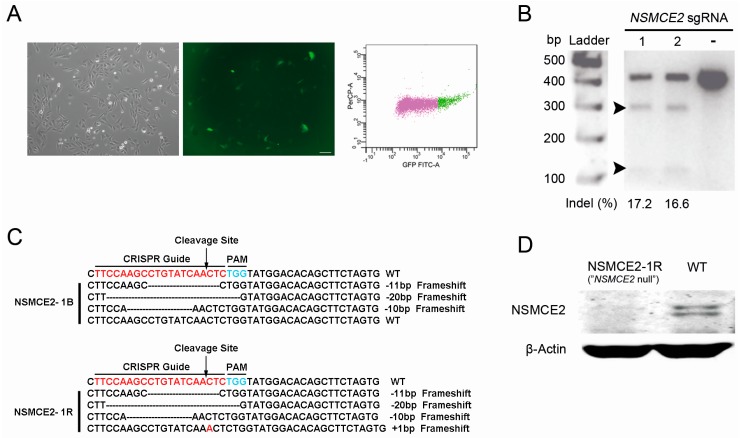
CRISPR-Cas9-mediated targeting of *NSMCE2*: (**A**) Left panel, transfected U2OS cells (GFP^+^) under bright field and fluorescence; right panel, FACS enrichment of GFP^+^ cells. Bar = 50 µm; (**B**) Expected cleavage bands (approximately 304 and 113 bp, arrowheads) generated by Surveyor nuclease digestion. For negative control (−) transfection of the pX458 plasmids without *NSMCE2* sgRNA was performed; (**C**) Sequencing analysis for characterization of the CRISPR-Cas9-induced frameshift mutations. Red letters represent the 20-nt targeting sequences, while blue letters refer to the protospacer-adjacent motif (PAM); (**D**) Western blot analysis of the NSMCE2 protein in the final *NSMCE2* null and WT cells. β-Actin was used as a loading control.

In order to investigate the role of the SMC5/6 complex during different cellular processes such as DNA repair, we used the novel CRISPR-Cas9 system to generate cells lacking a fully functional SMC5/6 complex. U2OS cells were transfected with constructed CRISPR plasmids (pX458) to target *SMC6* or *NSMCE2*. One day after transfection, GFP^+^ cells harboring CRISPR plasmids were sorted by fluorescence-activated cell sorting (FACS) and then cultured for five days (Figure 1A). To verify the occurrence of genome mutations in the sorted cell fractions, a Surveyor assay was performed based on PCR amplicons of the genomic DNA region around the targeting sites. Targeting of *NSMCE2*, but not *SMC6*, yielded fragments with the expected sizes after Surveyor nuclease digestion, indicating successful genome editing. The insertion/deletion (indel) occurrence brought by two individual single-guide RNAs (sgRNAs) targeting *NSMCE2* was 17.2% and 16.6%, respectively (Figure 1B). To derive a monoclonal knockout cell line, FACS was conducted to deposit single GFP^+^ cells into 96-well plates. Single cells were then expanded for one to two months. Consistent with the results of Surveyor assay, all single cell-derived colonies appeared wild type for *SMC6* after Sanger sequencing. In addition, for *NSMCE2*, we did not achieve complete *NSMCE2*-knockout after propagation. However, one monoclonal cell population (*NSMCE2*-1B) showed only one remaining wild type *NSMCE2* allele, which was effectively mutated after a second round of transfection and single cell sorting using the *NSMCE2*-1B clone, resulting in the generation of a complete *NSMCE2* null cell line (*NSMCE2*-1R, Figure 1C). Both Sanger sequencing and Western blot analysis showed the full null mutations in *NSMCE2*-1R cells (Figure 1C,D). Subsequently, by sequencing the 10 top-ranking potential off-target sites in the established *NSMCE2* null cell line (Table S1), no off-target alterations were detected.

### 2.2. Characterization of NSMCE2 Null Cells

**Figure 2:**
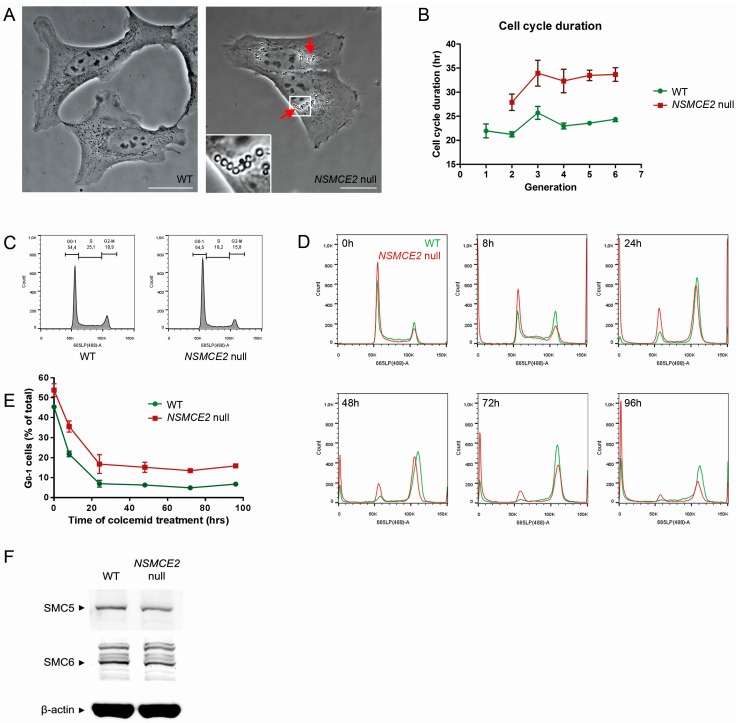
Analysis of *NSMCE2* null cell growth characteristics: (**A**) Phase contrast images of WT and *NSMCE2* null cells. The latter shows a large number of vacuoles in the cytoplasm (arrow). Bar = 20 µm; (**B**) Average cell cycle duration of WT and *NSMCE2* null cells over multiple generations observed by live cell imaging. Data are presented as mean ± standard error of mean (SEM), *n* = 3; (**C**) Cell cycle phase distribution analysis of WT and *NSMCE2* null cells by DNA histograms shows a 10% increase *NSMCE2* null cells in G_0-1_; (**D**) G_0-1_ phase depletion by colcemid treatment. WT and *NSMCE2* null cells were treated with colcemid for 0–96 h. In *NSMCE2* null cells, a fraction of cells remained in G_0-1_ even after 96 h; (**E**) Quantification for depletion of G_0-1_ cells with the time of colcemid treatment. Data are presented as mean ± SEM, *n* = 3; (**F**) Western blot analysis of SMC5 and SMC6 proteins in WT and *NSMCE2* null cells. β-Actin was used as a loading control.

Morphologically, *NSMCE2* null cells generally resemble WT cells, although *NSMCE2* null cells clearly show more vacuoles, indicating increased cellular stress in the absence of NSMCE2 (Figure 2A). In addition, time-lapse imaging revealed a significant 1.37-fold increase in the cell cycle duration of *NSMCE2* null cells (Figure 2B). When investigating the distribution of cells among different cell cycle phases, the DNA histogram of *NSMCE2* null cells showed a recurring increase of approximately 10% in G_0-1_ phase compared to WT (Figure 2C). To investigate whether all of the *NSMCE2* null cells participate in the cell cycle, we treated WT and *NSMCE2* null cells with the M-phase blocking agent colcemid [29]. Although both WT and *NSMCE2* null cells showed a rapid depletion of G_0-1_ cells after colcemid treatment (Figure 2D,E), which is in accordance with the rapid cycling nature of U2OS cells, there were always ~10% more *NSMCE2* null cells remaining in G_0-1_, and even after 96 h, a clear subpopulation of 16% remained (Figure 2D,E), indicating that these cells do not participate in the cell cycle. Protein levels of SMC5 and SMC6 were not evidently affected by the absence of NSMCE2 (Figure 2F).

### 2.3. Irradiation-Induced SMC6 Foci Formation Occurs Independent of NSMCE2

**Figure 3:**
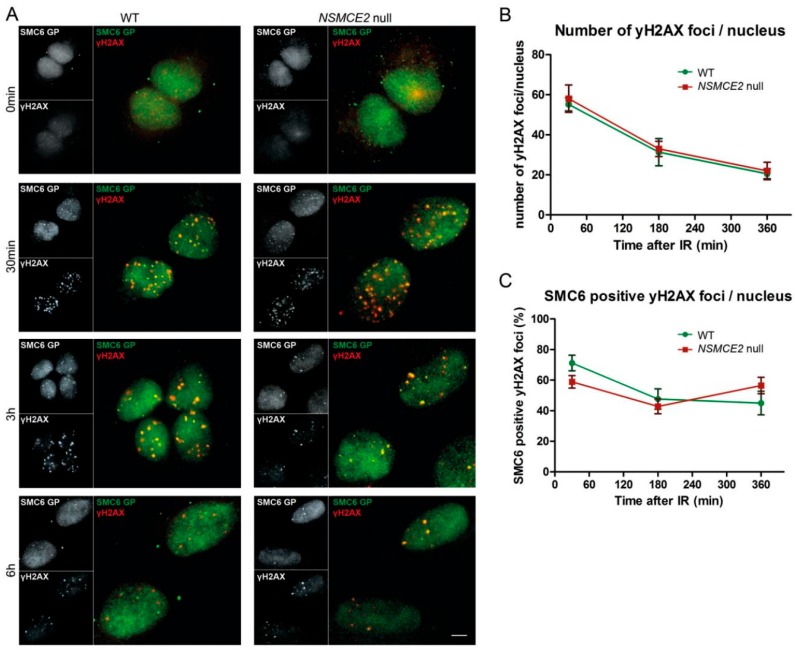
Ionizing irradiation (IR)-induced double-strand break (DSB) foci formation in the absence of NSMCE2. (**A**) WT and *NSMCE2* null cells were subjected to 1 Gy of IR, fixed at different time points post IR (0 min: immediately after IR) and stained for γH2AX (a marker for DNA damage, red) and SMC6 (green). SMC6 localized to sites of DNA damage in both WT and *NSMCE2* null cells. Bar = 5 μm; (**B**) Quantification of the average number of γH2AX foci in each cell. Data are presented as mean ± SEM, *n* = 3; (**C**) Quantification of the average number of γH2AX foci that overlap with a SMC6 focus. Data are presented as mean ± SEM, *n* = 3.

Because NSMCE2 is reported to be essential for the SMC5/6 function in the repair of DNA DSBs [30,31,32,33,34], we performed immunocytochemical stainings (ICC) for SMC6 on WT and *NSMCE2* null cells at different time points after exposure to 1 Gy of ionizing irradiation (IR) (Figure 3A). Indeed, both in WT and *NSMCE2* null cells, IR induced SMC6 foci that co-localize with DSBs (marked by γH2AX foci) and that gradually decrease post irradiation. This expression pattern of SMC6 was similar for both WT and *NSMCE2* null cells. After we generated and applied a MatLab image analysis script that objectively isolates and quantifies the number of SMC6 and γH2AX foci in each cell, we found no difference in average number of γH2AX foci per nucleus at the chosen time points, indicating that both cell lines process DSBs in a similar fashion (Figure 3B). Importantly, not all γH2AX foci were represented by a SMC6 focus (Figure 3A). We therefore also determined the percentage of γH2AX foci positive for SMC6. We found that roughly 50% of the DSB sites were positive for SMC6 in both cell lines, indicating that the accumulation of SMC6 to sites of DSB damage is equally efficient in WT and *NSMCE2* null cells (Figure 3C).

### 2.4. More Etoposide-Induced Double-Strand Break (DSB) Formation without NSMCE2

**Figure 4:**
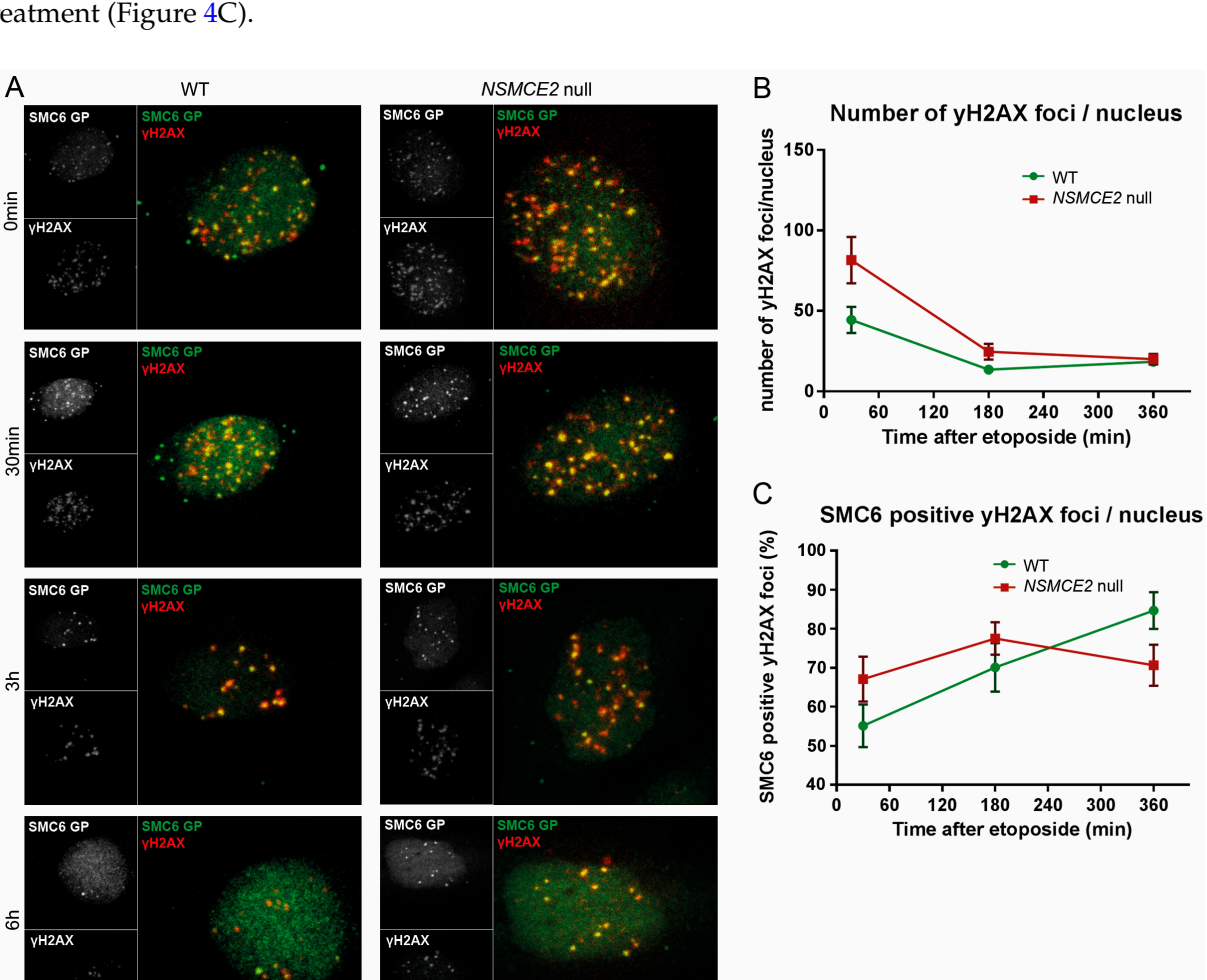
Etoposide-induced DSB foci formation in the absence of NSMCE2. (**A**) WT and *NSMCE2* null cells were subjected to 3 µM etoposide for 1 h, fixed at different time points post treatment (0 min: immediately after etoposide treatment) and stained for γH2AX (a marker for DNA damage, red) and SMC6 (green). SMC6 localized to sites of DNA damage in both WT and *NSMCE2* null cells. Bar = 5 μm; (**B**) Quantification of the average number of γH2AX foci in each cell. Significantly more γH2AX foci were formed in *NSMCE2* null cells. Data are presented as mean ± SEM, *n* = 3; (**C**) Quantification of the average number of γH2AX foci that overlap with a SMC6 focus. Data are presented as mean ± SEM, *n* = 3.

Next we interrogated whether the absence of NSMCE2 would influence the repair of etoposide-induced DNA damage. The cells were exposed to etoposide, a cytotoxic agent that acts by forming a complex with the DNA and the topoisomerase II enzyme [35,36]. In normal conditions, type II topoisomerase releases superhelical stress and untangles chromosomes by creating transient DSBs, through which an unbroken DNA helix is transferred before ligation of the break, thereby averting incidents such as stalled replication forks or replication-induced joint molecules caused by DNA supercoiling [37]. Because etoposide prevents re-ligation of the DNA strands after transient DSBs, etoposide treatment will eventually lead to an increase of permanent DSBs [35,36]. Indeed, when exposing the WT and *NSMCE2* null cells to etoposide, DSBs (marked by γH2AX) became readily discernible (Figure 4A). However, in contrast to IR, the number of etoposide-induced DSBs increased significantly in the absence of NSMCE2 (*p* < 0.02 after 30 min and *p* < 0.03 after 3 h, Figure 4B). Similar to IR, not all etoposide-induced DSB foci contained SMC6, and the percentage of DSB foci containing SMC6 showed no significant difference between WT and *NSMCE2* null cells after etoposide treatment (Figure 4C).

### 2.5. Absence of NSMCE2 Affects Survival upon Etoposide-Induced DSBs

**Figure 5:**
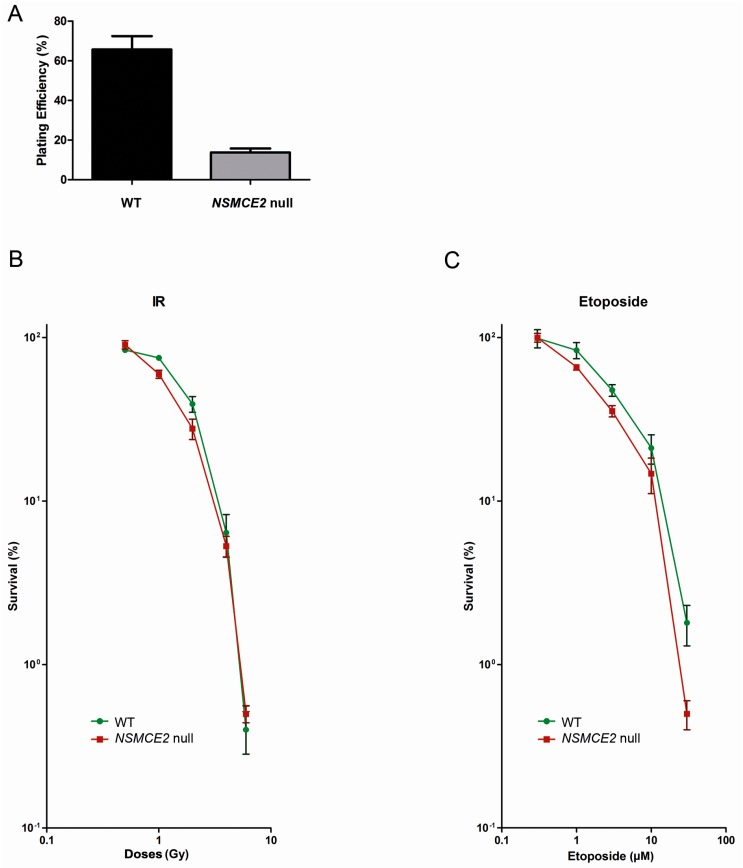
Absence of NSMCE2 affects survival upon etoposide-induced DSBs. (**A**) Plating efficiency of WT and *NSMCE2* null cells during clonogenic assays. The survival capacity of plated cells under non-challenged conditions was reduced in *NSMCE2* null cells compared to WT. Data are presented as mean ± SEM, *n* = 8; (**B**) Clonogenic assay after increasing doses of IR. *NSMCE2* null cells seemed to be slightly more sensitive to IR than WT cells (*p* > 0.05). Data are presented as mean ± SEM, *n* = 3; (**C**) Clonogenic assay after increasing doses of etoposide. *NSMCE2* null cells were significantly more sensitive to etoposide than WT cells. Data are presented as mean ± SEM, *n* = 3.

To measure the role of NSMCE2 in survival upon IR- or etoposide-induced DSBs, we subjected both *NSMCE2* null and WT cells to a clonogenic assay [38]. Firstly, the plating efficiency, i.e., the percentage of plated single cells that develop into a cell colony of at least 50 cells determined in control conditions, was over three-fold lower in *NSMCE2* null than in WT cells (average of 25% compared to 75%, respectively) (Figure 5A). Interestingly, the relative survival when cells were exposed to increasing doses of IR (0–8 Gy) only showed a small difference between the WT and *NSMCE2* null cells, with the latter being slightly more sensitive (not statistically different though, Figure 5B). However, when exposed to 1 h of increasing doses of etoposide, *NSMCE2* null cells showed a clearly reduced viability compared to WT cells (*p* < 0.05 in the case of 30 µM, Figure 5C).

### 2.6. The SMC5/6 Complex Physically Interacts with Topoisomerase II α (TOP2A)

**Figure 6:**
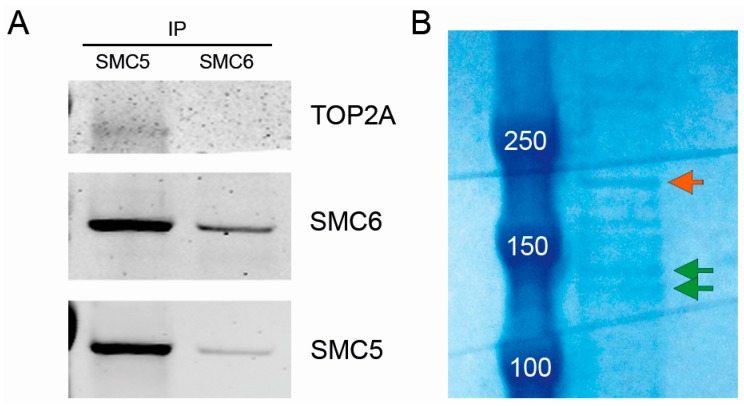
The SMC5/6 complex physically interacts with TOP2A. (**A**) Immunoprecipitations (IPs) and Western blot analysis of SMC5 and SMC6. Both SMC5 and SMC6 co-immunoprecipitated with each other. Additionally, TOP2A co-immunoprecipitated with SMC5; (**B**) For IP followed by mass spectrometry, Abcam rabbit anti-SMC6 was used. SMC6-IP material was loaded on a 4%–12% bis-tris gradient gel and stained with coomassie blue. Arrows indicate the bands that were isolated for mass spectrometry analysis (green: potential SMC5/6 proteins; orange: potential TOP2A protein).

To validate that the SMC5/6 complex is indeed involved in topoisomerase II-mediated relief of topological stress, we performed immunoprecipitations (IPs) using anti-SMC5 and SMC6 antibodies. Both SMC5 and SMC6 clearly co-immunoprecipitated with each other, and TOP2A clearly co-immunoprecipitated with SMC5 (Figure 6A), suggesting that the three proteins are physically linked. To unequivocally establish the physical interaction among these proteins, we conducted an additional IP with another antibody Abcam rabbit anti-SMC6, followed by mass spectrometry of the bands that could represent SMC5/6 and TOP2A (Figure 6B). We found that the band we presumed to represent full length SMC6 indeed contained the SMC6 protein. Not unexpectedly, because SMC5 and SMC6 are physically linked in the SMC5/6 complex, and SMC5 and SMC6 have equal sizes, we additionally identified the SMC5 protein to be present at the same height (Figure 6B). Interestingly, the lower running band (Figure 6B), which has been suggested to represent SMC6 lacking posttranslational modifications [9,10,39], was convincingly identified as SEC23IP, a protein previously shown to be involved in spermiogenesis [40]. Finally, we investigated the largest band (approximately 175 kDa) that could potentially represent TOP2A and that was also pulled down in this SMC6 IP (Figure 6B). Of the 23 identified peptides, 3 are homologous between TOP2A and TOP2B, and 20 are unique to TOP2A. Because no specific peptides for TOP2B were identified we conclude that this band indeed represented the protein TOP2A (Data S1–S3).

## 3. Discussion

To study the SMC5/6 complex in an experimental setup, we used the novel CRISPR-Cas9 system to target the *SMC6* and *NSMCE2* genes that encode the SMC6 and NSMCE2 subunits of the SMC5/6 complex, respectively. For *NSMCE2*, we did not get a complete knockout after the first round of transfection and monoclonal isolation. The results were not unexpected, given that U2OS is a cell line with chromosome counts in the hypertriploid range, and that generating a true knockout is technically challenging since it requires disruption of all functional copies of the gene. Consequently, a second round of gene targeting and single cell expansion was performed. Eventually we established a bona fide cell line devoid of *NSMCE2*. In the case of *SMC6*, however, we were unable to generate any CRISPR-modified cells. Previous papers show that ablation of *SMC6* resulted in embryonic lethality in mice [26] and that conditional knockout of *SMC5* in mouse embryonic stem cells induced apoptosis [28]. Therefore, the failure to generate *SMC6*-deprived clones in our studies most likely reflects that SMC6 is also essential for cell survival in humans.

To minimize the CRISPR-Cas9-induced off-target effects, we selected sgRNAs with the highest specificity to coding exons of *SMC6* and *NSMCE2*. Later, we analyzed the 10 top-ranking potential off-target sites in the established *NSMCE2* null cell line by sequencing, and detected no off-target mutations. The results are in line with recent papers showing low frequency of CRISPR-Cas9-induced off-target alterations in human cells [41,42]. Hence, although the possibility of off-target effects in modified cells could not be thoroughly excluded, the differential phenotypes between *NSMCE2* null and WT cells are thought to authentically mirror gene functions.

We found clear differences in growth characteristics of the *NSMCE2* null cells compared to WT cells. The mutated cells have a prolonged cell cycle, and a clear larger portion of the cells arrest in the G_0-1_ phase. Because this latter fraction is still equally present after an extensive time in culture and multiple passages, it must be continuously supplemented by cells exiting the cell cycle. Considering the differences in phenotypes, i.e., the presence of the G_0-1_-arrested cells, the overall slower growth rate and the reduced plating efficiency of *NSMCE2* null cells, it is plausible that the absence of NSMCE2 is not immediately lethal to the majority of the cells, but poses growth challenges in normal culture conditions that will ultimately arrest the cells.

These data are in line with a recent study performed in human breast cancer cells (MCF-7), in which the depletion of NSMCE2 by RNA interference (RNAi) caused a slower cell growth and increased percentage of G_1_ phase cells (70% vs. 55%–59% in control) [43]. Interestingly, depletion of NSMCE2 led to reduced levels of E2F1 protein and its downstream target genes that are required for G_1_-S transition. This decreased growth rate was rescued by ectopic expression of Flag-NSMCE2 but not its SUMO ligase-inactive mutant, suggesting that the SUMO ligase activity of NSMCE2 ensures proper G_1_-S transition in these human cancer cells [43].

Although NSMCE2 is frequently linked to the DNA repair function of the SMC5/6 complex [30,31,32,33,34,44,45], the survival capacity of *NSMCE2* null cells is only slightly affected after increasing doses of IR. Likewise, IR-induced DSBs marked by γH2AX appear with similar kinetic properties in WT and *NSMCE2* null cells. We therefore conclude that NSMCE2 is not crucial for the repair of IR-induced DSBs. Nevertheless, the recruitment of SMC6 to DSBs early post irradiation suggests that the SMC5/6 complex is involved in the early repair of DSBs. In addition, *NSMCE2* null cells did not display a differential survival rate in response to cisplatin, a cytostatic agent causing DNA adducts and crosslinks that are generally repaired by nucleotide excision repair (NER, Figure S1).

In contrast to the effects of IR or cisplatin, exposure to increasing doses of the topoisomerase inhibitor etoposide does impair the survival capacity of *NSMCE2* null cells. In normal conditions, type II topoisomerase releases superhelical stress and untangles chromosomes by creating transient DSBs, through which an unbroken DNA helix is transferred before ligation of the break, in order to unwind the DNA double helix to prevent supercoiling [37,46]. Etoposide acts as a cytotoxic agent by forming a complex with the DNA and the topoisomerase II enzyme, thereby preventing re-ligation of the DNA strands after transient DSBs. Thus, etoposide treatment will ultimately lead to an increase of permanent DSBs [35,36]. The increased sensitivity to etoposide of *NSMCE2* null cells is intriguing, especially in the light of the interaction between SMC5/6 and TOP2A found in this study. Interplay between TOP2A and the SMC5/6 complex has been suggested by several studies in mouse and yeast [13,47,48,49,50], but physical interaction between them is not confirmed. Recently, it has been reported that Smc5/6 immunoprecipitated with the type II topoisomerase in budding yeast [51]. Here, we for the first time unequivocally demonstrate that TOP2A is indeed associated with the SMC5/6 complex in human cells. The absence of a detectable band for TOP2A in the SMC6 guinea pig (GP) IP is most likely due to the lower efficiency of the SMC6 GP antibody, which is supported by the observation that the SMC5 IP generated more SMC6 protein than the SMC6 GP IP.

Topological tension is conceived when DNA molecules become supercoiled, for instance preceding an advancing replication fork during chromosome duplication. This positive supercoiling has to be removed in order for the replication fork to proceed. One mechanism to avoid accumulation of supercoiled DNA ahead of the fork is to allow the replication fork to proceed in a rotating manner following the turn of the helix. This will indeed prevent the accumulation of supercoils ahead of the fork, but will simultaneously induce the formation of sister chromatid intertwinings (SCI) behind the fork. Another way to release the supercoiling is through topoisomerases, the enzymes creating single-strand nicks and double-strand breaks. In this case TOP1 and TOP2 nick the DNA double helix ahead of the fork, thereby allowing the unwinding of the supercoiled helix and the release of topological tension. Because topological tension increases with the length of the chromosome, topoisomerases are supposed to be more important to replication of longer chromosomes. Indeed, budding yeast cells lacking functional topoisomerase I show a length-dependent delay in replication [52]. In line with the observed interaction between SMC5/6 and TOP2A, the association of budding yeast Smc5/6 with chromosomes during S-phase is also linearly correlated with chromosome length, indicating that Smc5/6 somehow measures chromosome length, probably by sensing topological tension [52,53]. Moreover, Smc5/6 seems to also play a role in topological strain release, since budding yeast cells lacking functional Smc6 or Nse2 show a delay in replication similar to Top2 mutants [52,53]. Our own data, showing that inhibition of topoisomerase activity has a more profound effect on cells harboring an impaired SMC5/6 complex, together with physical interaction between the SMC5/6 complex and TOP2A, further corroborate the presumed co-operation between TOP2A and SMC5/6 at replication forks.

Considering the two mechanisms of tension release, SMC5/6 could function both before and after the replication fork. Ahead of the fork, SMC5/6 could be responsible for the correct repair of the TOP2A-induced DSBs. When the ligase function of TOP2A is inhibited by etoposide, re-initiation of replication might rely more on SMC5/6, which will be challenged when NSMCE2 deletion impairs SMC5/6 function. On the other side of the fork, SMC5/6 might be required to stabilize the SCIs, as proposed previously [52]. In this model, SMC5/6 associates to SCIs, thereby fixating them and allowing fork rotation, and reducing topological tension. In parallel, budding yeast Smc5/6 is also involved in the actual resolving recombination intermediates in order to prevent toxic chromosome structures [54]. In addition, budding yeast Top2 is also essential for the removal of SCIs that would otherwise lead to segregation errors during the subsequent M-phase [55,56,57]. Since both induction and removal of SCIs involves transferring one DNA double helix through another via transient formation and repair of a DSB [46], it is likely that SMC5/6 is also working together with TOP2A at the level of SCIs.

Furthermore, in budding yeast, Top2 activity relies on sumoylation and failure to sumoylate Top2 disrupts the ability of Top2 to separate replicated chromosomes [58]. Of note, human TOP2 is also found conjugated to SUMO [59]. However, RANBP2 seems to be the major SUMO E3 ligase for TOP2A in mice [60], and a mutation compromising NSMCE2 sumoylation activity does not affect murine lifespan [27]. Nevertheless, we cannot rule out that SMC5/6 function at the replication fork might involve NSMCE2-mediated sumoylation of TOP2A, which could explain the interaction between SMC5/6 and TOP2A and the effects seen in *NSMCE2* null cells.

Our findings, demonstrating a physical interaction between SMC5/6 and TOP2A and an increased sensitivity of *NSMCE2* null cells to etoposide, suggest that the SMC5/6 complex helps to resolve topological stress. Since this physical interaction is even present in cells that are not challenged by IR or cytotoxic agents, SMC5/6 and TOP2A seem already to function together during S-phase under normal non-challenged circumstances. In this respect, it is plausible that the fraction of *NSMCE2* null cells arresting in G_0-1_ phase is actually a representation of cells with stalled or collapsed replication forks very early in the replication process, which occurs naturally, yet cannot be resolved properly due to the lack of NSMCE2. While in WT cells these replication forks are normally repaired and restarted by SMC5/6, the absence of NSMCE2 first induces a delay in early replication progression, explaining the increased cell cycle duration of *NSMCE2* null cells. Subsequently, residual repair defects may trigger cells to eventually arrest in G_0_. In this respect, several studies in yeast have suggested that Smc5/6-mutated cells will undergo cell division despite the presence of chromosomal abnormalities caused by defective DNA repair mechanisms [30,47,50,61,62]. However, over time, the amount of chromosomal abnormalities within a cell will accumulate, eventually leading to cell cycle arrest.

In conclusion, in the light of current literature and the data we present here, we propose that the SMC5/6 complex functions in resolving TOP2A-mediated recombination intermediates endogenously generated early during DNA replication in human cells.

## 4. Materials and Methods

### 4.1. Cells and Culture

U2OS cells were cultured at 37 °C and 10% CO_2_ in Dulbecco’s modified Eagle’s medium (DMEM; (high glucose, pyruvate, l-glutamine); Thermo Fisher Scientific, Waltham, MA, USA) supplemented with 10% Fetal Calf Serum (FCS), penicillin (100 U/mL) and streptomycin (100 U/mL).

### 4.2. Design of Single-Guide RNAs (sgRNAs) and Construction of the CRISPR-Cas9 Plasmids

An online CRISPR-Cas9 Design Tool provided by Zhang’s lab (http://crispr.mit.edu) was exploited to identify the targeting sequences. The 20-nt targeting sequences preceding 5′-NGG (the protospacer-adjacent motif, PAM), locating in early and conserved coding exons among transcript variants, and with least predicted off-target sites in human genome were selected. To target *SMC6* and *NSMCE2* in U2OS cells, two sgRNAs were designed for each gene: 5′-GGTGACGAAGACGAATGTAA-3′ (in exon 3) and 5′-ATGCTTGGACCTTTTAAGTT-3′ (in exon 4) for *SMC6*, 5′-TTCCAAGCCTGTATCAACTC-3′ and 5′-AGCCTGTATCAACTCTGGTA-3′ (both in exon 3) for *NSMCE2*. The corresponding sense and antisense strands of oligos were purchased from Sigma-Aldrich (St. Louis, MO, USA). The CRISPR plasmids pSpCas9(BB)-2A-GFP (pX458) were obtained from Addgene (Addgene plasmid 48138). The pX458 plasmids were digested with FastDigest BbsI (Fermentas, Waltham, MA, USA). The oligos were then annealed and cloned into digested pX458 according to the protocol described by Ran et al. [63].

### 4.3. Transfection of U2OS Cells with CRISPR Plasmids

The constructed pX458 plasmids were transfected into U2OS cells with the 4D-Nucleofector System (Lonza, Basel, Switzerland). For each nucleofection reaction, approximately 500 ng plasmids were transfected into 100,000 U2OS cells using program CM-104 and the SE Cell Line 4D-Nucleofector X Kit S (Lonza), according to the manufacturer’s instructions. One day after nucleofection, the top 5%–10% GFP^+^ U2OS cells were separated by FACS with a BD FACSAria cell sorter. In order to derive a stable *NSMCE2*-deficient cell line from single U2OS cells, FACS was conducted to deposit single GFP^+^ cells into 96-well plates (one cell per well).

### 4.4. Surveyor Assay for Verification of Genome Editing

Genomic DNA of transfected U2OS cells was extracted with QIAamp DNA Mini Kit (Qiagen, Hilden, Germany), according to the protocol provided by the manufacturer. The genomic region (417 bp) containing both mutation sites in exon 3 of *NSMCE2* was amplified by PCR with Herculase II fusion polymerase (Agilent Technologies, Santa Clara, CA, USA). The forward and reverse primers used for PCR were 5′-AATTTCAAGATGCCAGGACGT-3′ and 5’-GGATCTTCAAATCTTTGCCCAT-3′, respectively. PCR products were purified by QIAquick PCR Purification Kit (Qiagen). Genomic modifications in the amplified region were then detected with Surveyor Mutation Detection Kit (Integrated DNA Technologies, Coralville, IA, USA), according to the manufacturer’s instructions. After Surveyor nuclease digestion, the PCR products were run on a 2% agarose gel with ethidium bromide (EB) for visualization. The insertion/deletion (indel) occurrence was estimated with the formula described by Ran et al. [63].

### 4.5. Sequencing Analysis of the Targeting Site in NSMCE2

Genomic DNA was extracted from each single cell-derived colonies, and the region flanking the targeting site was amplified by PCR with Easy-A High-Fidelity PCR Cloning Enzyme (Agilent Technologies). The primers used were identical to those for the Surveyor assay. After purification, the PCR products were cloned into TOPO TA cloning vectors (Thermo Fisher Scientific). The ligated vectors were transformed into One Shot TOP10 Chemically Competent *E. coli* (Thermo Fisher Scientific). For each reaction, forty colonies were randomly picked and subjected to Sanger-sequencing to analyze mutations from all alleles.

### 4.6. Off-Target Analysis in the NSMCE2-Devoid Cell Line

Genomic DNA was extracted from WT and *NSMCE2* null U2OS cells, respectively. To gain an overview of off-target effects in the established *NSMCE2* null cell line, the 10 top-ranking potential off-target sites provided by the online CRISPR Design Tool (http://crispr.mit.edu) were analyzed. The selected sites included those preceding 5′-NAG, the alternative PAM. The genomic regions flanking each potential off-target sites were amplified by PCR with Herculase II fusion polymerase (Agilent Technologies). The selected off-target sites and the corresponding PCR primers are depicted in Table S1. Purified PCR products were Sanger-sequenced for analysis of off-target effects.

### 4.7. Live Cell Microscopy

Cells were plated in multi-chambered cover-glass slides (Labtek II, Nunc) in a density of 1000 cells/cm^2^ and cultured overnight before starting imaging. Both U2OS WT and *NSMCE2* null cells were imaged at the same time, using a IRBE inverted phase contrast microscope and a N Plan Apo L 40×/0.55 Ph2 objective. Images were captured every 10 min for a total of 170 h, at 37 °C in an atmosphere containing 10% CO_2_. Medium was refreshed under the microscope. Cell cycle duration was determined by calculating the time between two cell divisions. Generations were aligned, in which we chose the third generation as the one in which the medium was refreshed, based on the WT cells.

### 4.8. Distribution of Cells over the Cell Cycle Phase

To determine the distribution of cells over the different cell cycle phases, colcemid (KaryoMAX Colcemid Solution; Thermo Fisher Scientific) was added to the culture medium to a final concentration of 0.1 µg/mL and DNA histograms were made after incubation. Cells were detached by 0.05% trypsin/EDTA (Thermo Fisher Scientific), pelleted in serum-containing medium and washed in PBS (Phosphate Buffered Saline). Cells were fixed and stored in 100% EtOH at 4 °C. On the day of FACS analysis, cells were pelleted by centrifugation and all EtOH was removed. Cells were resuspended in PBS and RNAse A (final 1 mg/mL; Roche, Basel, Switzerland) was added. After vortexing, propidium iodide (PI; final 25 µg/mL; Sigma-Aldrich) was added and the cell suspension was thoroughly vortexed again. Cells were incubated for 15 min at 37 °C, after which the cell suspension was transferred through a 21 G needle twice, in order to disrupt cell aggregates. DNA content was analyzed on a LSR Fortessa FACS analyzer (BD Biosciences, San Jose, CA, USA) using Diva^TM^ acquisition and analysis software. Figures were constructed using FlowJo X software.

### 4.9. Ionizing Irradiation (IR)

Exponentially growing cells were exposed to IR emitted by a ^137^Cs source (95% β-emission). For immunocytochemistry, cells received 1 Gy of IR. For clonogenic assays, cells received 0–8 Gy of IR.

### 4.10. Immunocytochemistry (ICC)

For ICC, cells were plated on glass coverslides overnight, after which they were fixed in 4% formaldehyde/PBS for 10 min at room temperature (RT). In the case of IR treatment, IR-treated cells (and their non-IR counterparts) were fixed at varying time points, ranging from 0 min to 6 h post IR. In the case of etoposide treatment, cells were incubated with 3 µM etoposide for 1 h at 37 °C/10% CO_2_. Cells were fixed at varying time points after removal of etoposide, ranging from 0 min to 6 h post etoposide treatment. After fixation, cells were permeabilized for 10 min at RT in PBST (0.25% Tween-20/PBS). Next, non-specific adhesion sites were blocked for 45 min at RT in PBST containing 1% bovine serum albumin (BSA). To visualize SMC6 and γH2AX, slides were incubated for 2 h at RT in primary antibodies guinea pig (GP) anti-SMC6 (1/200; custom made, peptide: KRPRQEELEDFDKDGDEDE) and mouse anti-γH2AX (1/10,000; 05-636, Merck Millipore, Darmstadt, Germany), diluted in 1% BSA/PBST. After incubation in corresponding secondary antibodies (Goat-anti-GP Alexa488, Goat-anti-Mouse Alexa555, respectively; all diluted 1/1000 in 1% BSA/PBST), slides were washed and counterstained with DAPI and mounted in Prolong Gold. Between all steps, except blocking and primary antibody incubation, 3 × 5 min washes in PBS were performed.

Widefield fluorescence microscopy images were acquired at RT using a Plan Fluotar 100×/1.30 oil objective on a Leica DM5000B widefield microscope equipped with a Leica DFC365 FX CCD camera. Images were analyzed using Leica Application Suite Advanced Fluorescence software. Figures were constructed using Adobe Photoshop CS5 version 12.0. Confocal images for subsequent quantification were acquired at RT using a Leica TCS SP8 SMD confocal microscope equipped with a 63×/1.40 HC Plan Apo oil CS2 objective (Leica, Wetzlar, Germany). For excitation of DAPI, a 405 nm UV Diode was used and for excitation of other fluochromes, the VIS Argon 470–670 nm White Light Laser (WLL) was used. Fluorescent signal was detected by PMTs and a HyD detector, and acquisition of the image (stacks) was performed using LAS AF X software.

### 4.11. Quantitative Imaging

In order to enable identical staining and acquisition conditions for all samples, four time points were chosen for quantitative imaging, 0 min, 30 min, 3 h and 6 h post IR or etoposide treatment. Confocal image stacks were acquired using the following settings: resonant scan = on; galvo flow and bidirectional X = off; line average = 4; acquisition = between lines; field of view = 792 × 792 (zoom = 5.0); Z-step size = 0.20 µm; stack size = 8 µm total (42 steps). One pixel = 47 × 47 × 200 nm (XYZ).

Images were deconvolved using Huygens Essential software, with a maximum number of iterations of 40, and a SNR setting of 12 (green channel) or 10 (red channel). By visual inspection, cells with at least two clear SMC6 foci were identified for further analysis with MatLab software. Using MatLab software, we isolated the γH2AX foci with a minimal size of 50 pixels (0.022 µm^3^) present in the nucleus. Next, the separate SMC6 foci were isolated (minimal size 10 pixels), and the amount of γH2AX foci overlapping with a SMC6 focus was determined. Statistical significance was determined by applying the Student’s *t*-test (two-tailed, unpaired).

### 4.12. Clonogenic Assay

Clonogenic assays were performed as described previously [38]. Four hours after plating, cells were exposed to 0–8 Gy of IR or incubated for 1 h at 37 °C in 0–30 μM etoposide. In each experiment, each dose was administered to 2 different cell densities. Experiments were repeated at least 3 times. Survival capacity was calculated relative to the non-treated control condition. Statistical significance was determined by applying the Student’s *t*-test (one-tailed, paired).

### 4.13. Protein Isolation

Cells were detached, washed in PBS and pelleted by centrifugation. These cell pellets were either snapfrozen in liquid nitrogen and stored or lysed directly. Cells were lysed in RIPA buffer containing 1× PIC and 1× PhosSTOP (Roche) for 1 h on ice. The lysate was centrifuged (16,000 rcf, 10 min, 4 °C) to clarify the extract.

### 4.14. Western Blot Analysis

Western blot analysis of cell lysates was performed as previously described [10], using the primary antibodies: SMC6 GP (1/200; custom made), SMC5 (1/1000; A300-236A, Bethyl Laboratories, Montgomery, TX, USA), NSMCE2 (1/500; NBP1-76263, Novus Biologicals, Littleton, CO, USA), TOP2A (1/1,000; TG2011-1, TopoGEN, Buena Vista, CO, USA) and β-actin (1/5000; A1978, Sigma-Aldrich).

### 4.15. Immunoprecipitation (IP)

IP was performed on lysed cells, using Dynabeads Protein A (Thermo Fisher Scientific). Per IP, 1 × 10^6^ cells and 50 µL dynabeads were used. Dynabeads were resuspended in AB Binding & Washing buffer containing 2 µL anti-SMC6 GP (custom made) or anti-SMC5 (A300-236A, Bethyl Laboratories) antibody and incubated for 30 min with rotation at RT. Using a magnet, the supernatant was removed, and the beads were washed by resuspension in AB Binding & Washing buffer. After the removal of the buffer, the beads were incubated in the cell lysate for 30 min with rotation at RT, after which the supernatant was collected and the beads were washed. Elution of the precipitated antigen was achieved after resuspension of the beads in RIPA buffer containing 1× PIC and 1× PhosSTOP, addition of LDS Sample Buffer and Sample Reducing Agent and heating of the sample for 10 min at 70 °C. In preparation for Western blot analysis, the supernatant was also supplemented with LDS Sample Buffer and Sample Reducing Agent and heated for 10 min at 70 °C.

### 4.16. Mass Spectrometry

Abcam rabbit anti-SMC6 (ab18039, Abcam, Cambridge, UK) was used for IP followed by mass spectrometry of the several bands detected by the antibody in U2OS cells, following the protocol described above. A total of approximately 7 × 10^6^ cells, 100 µL dynabeads and 5 µL SMC6 Abcam antibody were used. All immunoprecipitated material was loaded on a single lane of a 4%–12% bis-tris gradient gel (Thermo Fisher Scientific). After running, the gel was washed 3 times 10 min in H_2_O to remove SDS, and subsequently stained in a colloidal coomassie solution (PageBlue Protein Staining Solution; 24620, Thermo Fisher Scientific) for 1 h at RT, after which the excess of staining was washed away with H_2_O. Gels were stored in 1% acetic acid/H_2_O at 4 °C. Protein bands of interest were excised, alkylated and subjected to tryptic digestion according to standard protocols. Further mass spectrometry analysis was performed as described previously [64].

## Supplementary Materials

Supplementary materials can be found at www.mdpi.com/1422-0067/17/11/1782/s1.

## Abbreviations

SMC	Structural Maintenance of Chromosomes
NSMCE	Non-SMC Element
DSB	DNA double-strand break
IR	Ionizing Radiation
TOP	Topoisomerase
SCI	Sister chromatid intertwining
