# Supplementary material for: Non-SMC Element 2 (NSMCE2) of the SMC5/6 Complex Helps to Resolve Topological Stress
Source: Int J Mol Sci. 2016 Oct 26;17(11):1782. doi: 10.3390/ijms17111782 (PMC5133783; doi:10.3390/ijms17111782)
Supplement: Supplementary file 1 [file ijms-17-01782-s001.zip › ijms-142388-Supplementary Materials/ijms-142388-Supplementary Materials.pdf]

Supplementary Materials: Non-SMC Element 2 (NSMCE2) of the SMC5/6 Complex Helps to Resolve Topological Stress

Dideke E. Verver, Yi Zheng, Dave Speijer, Ron Hoebe, Henk L. Dekker, Sjoerd Repping, Jan Stap and Geert Hamer

Table S1. The selected off-target sites and the corresponding PCR primers.

Potential Off-Target Sites	Genomic Loci (GRCh38/hg38)	Mis Matches	PCR Primers
ACCCAAATCTGTATCA ACTCAGG	62065416-62065438, chromosome 11	1, 2, 7, 8	F: GTCCCTCCATCTTGGTGCCT<br>R: GGGTCTTTGCTGCCTGTGA
CTCTTATCCTGTATCAA CTCAAG	43274942-43274964, chromosome 2	1, 4, 5, 7	F: CACTGACAACAGGCATGAAAT<br>R: CTGGAGACTGAGGCAGGAGA
TTTCTATTCTGTATCAA CTCAAG	153197613-153197635, chromosome 7	3, 5, 7, 8	F: ATTGGGCCTTATGAACTGATTC<br>R: TGGTCTACGCAGGGTAAGGATA
TGCCAAGGCTGTATGA ACTCAGG	110987269-110987291, chromosome 2 (in gene)	2, 8, 15	F: ACTGCTTGGAACAGTGAACATG<br>R: GCTGAGACTGATGAGCGATAAA
TCCAAAGTCTGTATCA ACTTTAG	2809500-2809522, chromosome 8	2, 4, 8, 20	F: CAGCCAAACCATATCATTCTGT<br>R: TGTTTTCATGTTTGTGGCAGTG
TTCCAAGACTATATCA ACTAAAG	114757228-114757250, chromosome 11	8, 11, 20	F: GTATTGCAGCAAGCCATTACC<br>R: AAGAATCTGCTCTGGAGGGAG
TTCCTGGCCTGTATCA ACACAAG	52436381-52436403, chromosome 12	5, 6, 19	F: ACGGAACCAGGTGAAGGAA<br>R: TTGGCACTTGGAGCGGTAG
TTTGAAGTCTGTATCAT CTCAAG	104356169-104356191, chromosome 5	3, 4, 8, 17	F: CACTCTTACTTTGTTCCCCACA<br>R: CCTCACTTGCCTTGCCTATT
TACTAAACATGTATCA ACTCAGG	31343808-31343830, chromosome X	2, 4, 7, 9	F: GCTGGCGGGTGCAATTAGT<br>R: AGAGCAAGACCCTGACCCTAA
GCCCAAGCCAGTATCA CCTCAAG	128369750-128369772, chromosome 5	1, 2, 10, 17	F: CGGAAAGTGGGAGTAAGAAATC<br>R: GCTCTAATCACTGGCTATGCTAT

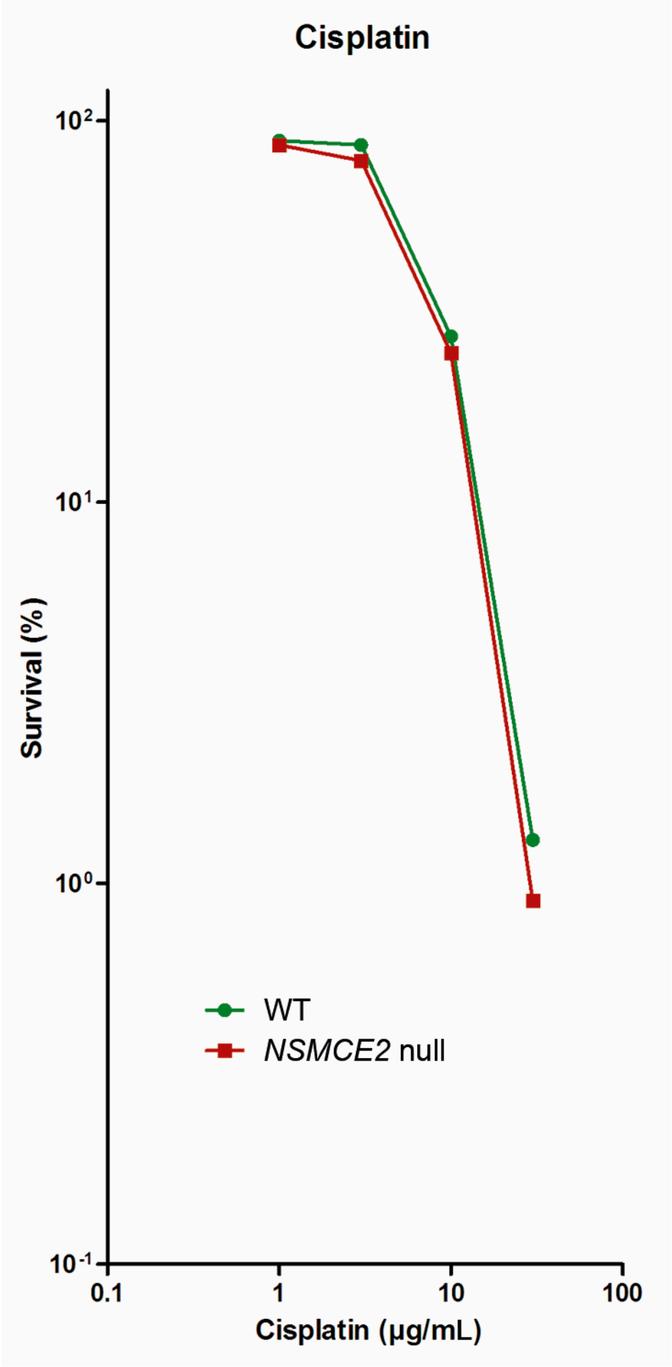

Figure S1. Clonogenic assay after increasing doses of cisplatin. No clear difference in sensitivity to cisplatin could be detected between WT and *NSMCE2* null cells.
