# Supplementary material for: Non-SMC Element 2 (NSMCE2) of the SMC5/6 Complex Helps to Resolve Topological Stress
Source: Int J Mol Sci. 2016 Oct 26;17(11):1782. doi: 10.3390/ijms17111782 (PMC5133783; doi:10.3390/ijms17111782)
Supplement: Supplementary file 1 [file ijms-17-01782-s001.zip › ijms-142388-Supplementary Materials/ijms-142388 Data S1-S3.zip!/ijms-142388 Data S1-S3/Supplementary Data 1.htm]

Mascot Search Results: TOP2A\_HUMAN


# MASCOT Search Results

## Protein View: TOP2A\_HUMAN

### DNA topoisomerase 2-alpha OS=Homo sapiens GN=TOP2A PE=1 SV=3

|  |  |
| --- | --- |
| Database: | SwissProt |
| Score: | 542 |
| Nominal mass (M_r_): | 175017 |
| Calculated pI: | 8.82 |
| Taxonomy: | Homo sapiens |

Sequence similarity is available as an NCBI BLAST search of TOP2A\_HUMAN against nr.

### Search parameters

|  |  |
| --- | --- |
| MS data file: | `ext1861_E7_300_RT.mgf` |
| Enzyme: | Trypsin: cuts C-term side of KR unless next residue is P. |
| Fixed modifications: | Carbamidomethyl (C) |
| Variable modifications: | Oxidation (M) |

### Protein sequence coverage: 21%

Matched peptides shown in ***bold red***.

|  |  |  |  |  |  |
| --- | --- | --- | --- | --- | --- |
| `1` | `MEVSPLQPVN` | `ENMQVNKIKK` | `NEDAKKRLSV` | `ERIYQKKTQL` | `EHILLRPDTY` |
| `51` | `IGSVELVTQQ` | `MWVYDEDVGI` | `NYREVTFVPG` | `LYKIFDEILV` | `NAADNKQRDP` |
| `101` | `KMSCIRVTID` | `PENNLISIWN` | `NGKGIPVVEH` | `KVEKMYVPAL` | `IFGQLLTSSN` |
| `151` | `YDDDEKKVTG` | `GRNGYGAKLC` | `NIFSTKFTVE` | `TASREYKKMF` | `KQTWMDNMGR` |
| `201` | `AGEMELKPFN` | `GEDYTCITFQ` | `PDLSKFKMQS` | `LDKDIVALMV` | `RRAYDIAGST` |
| `251` | `KDVKVFLNGN` | `KLPVKGFRSY` | `VDMYLKDKLD` | `ETGNSLKVIH` | `EQVNHRWEVC` |
| `301` | `LTMSEKGFQQ` | `ISFVNSIATS` | `KGGRHVDYVA` | `DQIVTKLVDV` | `VKKKNKGGVA` |
| `351` | `VKAHQVKNHM` | `WIFVNALIEN` | `PTFDSQTKEN` | `MTLQPKSFGS` | `TCQLSEKFIK` |
| `401` | `AAIGCGIVES` | `ILNWVKFKAQ` | `VQLNKKCSAV` | `KHNRIKGIPK` | `LDDANDAGGR` |
| `451` | `NSTECTLILT` | `EGDSAKTLAV` | `SGLGVVGRDK` | `YGVFPLRGKI` | `LNVREASHKQ` |
| `501` | `IMENAEINNI` | `IKIVGLQYKK` | `NYEDEDSLKT` | `LRYGKIMIMT` | `DQDQDGSHIK` |
| `551` | `GLLINFIHHN` | `WPSLLRHRFL` | `EEFITPIVKV` | `SKNKQEMAFY` | `SLPEFEEWKS` |
| `601` | `STPNHKKWKV` | `KYYKGLGTST` | `SKEAKEYFAD` | `MKRHRIQFKY` | `SGPEDDAAIS` |
| `651` | `LAFSKKQIDD` | `RKEWLTNFME` | `DRRQRKLLGL` | `PEDYLYGQTT` | `TYLTYNDFIN` |
| `701` | `KELILFSNSD` | `NERSIPSMVD` | `GLKPGQRKVL` | `FTCFKRNDKR` | `EVKVAQLAGS` |
| `751` | `VAEMSSYHHG` | `EMSLMMTIIN` | `LAQNFVGSNN` | `LNLLQPIGQF` | `GTRLHGGKDS` |
| `801` | `ASPRYIFTML` | `SSLARLLFPP` | `KDDHTLKFLY` | `DDNQRVEPEW` | `YIPIIPMVLI` |
| `851` | `NGAEGIGTGW` | `SCKIPNFDVR` | `EIVNNIRRLM` | `DGEEPLPMLP` | `SYKNFKGTIE` |
| `901` | `ELAPNQYVIS` | `GEVAILNSTT` | `IEISELPVRT` | `WTQTYKEQVL` | `EPMLNGTEKT` |
| `951` | `PPLITDYREY` | `HTDTTVKFVV` | `KMTEEKLAEA` | `ERVGLHKVFK` | `LQTSLTCNSM` |
| `1001` | `VLFDHVGCLK` | `KYDTVLDILR` | `DFFELRLKYY` | `GLRKEWLLGM` | `LGAESAKLNN` |
| `1051` | `QARFILEKID` | `GKIIIENKPK` | `KELIKVLIQR` | `GYDSDPVKAW` | `KEAQQKVPDE` |
| `1101` | `EENEESDNEK` | `ETEKSDSVTD` | `SGPTFNYLLD` | `MPLWYLTKEK` | `KDELCRLRNE` |
| `1151` | `KEQELDTLKR` | `KSPSDLWKED` | `LATFIEELEA` | `VEAKEKQDEQ` | `VGLPGKGGKA` |
| `1201` | `KGKKTQMAEV` | `LPSPRGQRVI` | `PRITIEMKAE` | `AEKKNKKKIK` | `NENTEGSPQE` |
| `1251` | `DGVELEGLKQ` | `RLEKKQKREP` | `GTKTKKQTTL` | `AFKPIKKGKK` | `RNPWSDSESD` |
| `1301` | `RSSDESNFDV` | `PPRETEPRRA` | `ATKTKFTMDL` | `DSDEDFSDFD` | `EKTDDEDFVP` |
| `1351` | `SDASPPKTKT` | `SPKLSNKELK` | `PQKSVVSDLE` | `ADDVKGSVPL` | `SSSPPATHFP` |
| `1401` | `DETEITNPVP` | `KKNVTVKKTA` | `AKSQSSTSTT` | `GAKKRAAPKG` | `TKRDPALNSG` |
| `1451` | `VSQKPDPAKT` | `KNRRKRKPST` | `SDDSDSNFEK` | `IVSKAVTSKK` | `SKGESDDFHM` |
| `1501` | `DFDSAVAPRA` | `KSVRAKKPIK` | `YLEESDEDDL` | `F` |  |

Unformatted sequence string: 1531 residues (for pasting into other applications).

Residue Number

Increasing Mass

Decreasing Mass

| Query | Start | – | End | Observed | Mr(expt) | Mr(calc) | Delta | M | Score | Expect | Rank | U | Peptide |
| --- | --- | --- | --- | --- | --- | --- | --- | --- | --- | --- | --- | --- | --- |
| 174 | 74 | – | 83 | 576.8400 | 1151.6654 | 1151.6227 | 0.0428 | 0 | 28 | 0.29 | 1Score **> 42** indicates **identity** Score **> 35** indicates **homology** | U | R.EVTFVPGLYK.I |
| 211 | 84 | – | 96 | 731.3990 | 1460.7834 | 1460.7511 | 0.0324 | 0 | 102 | 5.1e-08 | 1Score **> 42** indicates **identity** | U | K.IFDEILVNAADNK.Q |
| 136 | 169 | – | 176 | 491.7850 | 981.5554 | 981.4953 | 0.0601 | 0 | 34 | 0.065 | 1Score **> 42** indicates **identity** Score **> 35** indicates **homology** | U | K.LCNIFSTK.F |
| 120 | 177 | – | 184 | 455.7570 | 909.4994 | 909.4556 | 0.0439 | 0 | 42 | 0.015 | 1Score **> 42** indicates **identity** Score **> 36** indicates **homology** | U | K.FTVETASR.E |
| 180 | 192 | – | 200 | 585.7370 | 1169.4594 | 1169.4594 | 0.0001 | 0 | 31 | 0.022 | 1Score **> 42** indicates **identity** Score **> 27** indicates **homology** | U | K.QTWMDNMGR.A + 2 Oxidation (M) |
| 156 | 242 | – | 251 | 541.3090 | 1080.6034 | 1080.5564 | 0.0471 | 1 | 45 | 0.03 | 1Score **> 42** indicates **identity** | U | R.RAYDIAGSTK.D |
| 123 | 243 | – | 251 | 463.2600 | 924.5054 | 924.4552 | 0.0502 | 0 | 37 | 0.066 | 1Score **> 42** indicates **identity** Score **> 38** indicates **homology** | U | R.AYDIAGSTK.D |
| 144 | 269 | – | 276 | 517.7800 | 1033.5454 | 1033.4790 | 0.0664 | 0 | 22 | 0.55 | 1Score **> 43** indicates **identity** Score **> 32** indicates **homology** | U | R.SYVDMYLK.D + Oxidation (M) |
| 189 | 277 | – | 287 | 610.3060 | 1218.5974 | 1218.6092 | -0.0117 | 1 | 51 | 0.0069 | 1Score **> 42** indicates **identity** | U | K.DKLDETGNSLK.V |
| 226 | 307 | – | 321 | 813.9340 | 1625.8534 | 1625.8413 | 0.0121 | 0 | 61 | 7.1e-05 | 1Score **> 41** indicates **identity** Score **> 32** indicates **homology** | U | K.GFQQISFVNSIATSK.G |
| 209 | 325 | – | 336 | 694.3360 | 1386.6574 | 1386.7143 | -0.0569 | 0 | 94 | 3.5e-07 | 1Score **> 42** indicates **identity** | U | R.HVDYVADQIVTK.L |
| 130 | 379 | – | 386 | 480.7840 | 959.5534 | 959.4746 | 0.0789 | 0 | 26 | 0.43 | 1Score **> 43** indicates **identity** Score **> 35** indicates **homology** | U | K.ENMTLQPK.S |
| 133 | 379 | – | 386 | 488.7630 | 975.5114 | 975.4695 | 0.0419 | 0 | 27 | 0.46 | 1Score **> 43** indicates **identity** Score **> 36** indicates **homology** | U | K.ENMTLQPK.S + Oxidation (M) |
| 192 | 387 | – | 397 | 622.2920 | 1242.5694 | 1242.5551 | 0.0144 | 0 | 45 | 0.011 | 1Score **> 42** indicates **identity** Score **> 38** indicates **homology** | U | K.SFGSTCQLSEK.F |
| 81 | 419 | – | 425 | 400.7360 | 799.4574 | 799.4552 | 0.0023 | 0 | 30 | 0.66 | 1Score **> 42** indicates **identity** Score **> 41** indicates **homology** | U | K.AQVQLNK.K |
| 139 | 441 | – | 450 | 502.2390 | 1002.4634 | 1002.4366 | 0.0268 | 0 | 78 | 1.8e-05 | 1Score **> 43** indicates **identity** | U | K.LDDANDAGGR.N |
| 109 | 451 | – | 466 | 869.8890 | 1737.7634 | 1737.8091 | -0.0456 | 0 | 104 | 3.5e-09 | 1Score **> 42** indicates **identity** Score **> 32** indicates **homology** | U | R.NSTECTLILTEGDSAK.T |
| 170 | 467 | – | 478 | 564.8590 | 1127.7034 | 1127.6663 | 0.0372 | 0 | 68 | 0.00017 | 1Score **> 42** indicates **identity** | U | K.TLAVSGLGVVGR.D |
| 171 | 467 | – | 478 | 564.8590 | 1127.7034 | 1127.6663 | 0.0372 | 0 | 81 | 8.5e-06 | 1Score **> 42** indicates **identity** | U | K.TLAVSGLGVVGR.D |
| 221 | 500 | – | 512 | 773.3700 | 1544.7254 | 1544.7868 | -0.0613 | 0 | 56 | 0.00078 | 1Score **> 41** indicates **identity** Score **> 38** indicates **homology** | U | K.QIMENAEINNIIK.I + Oxidation (M) |
| 1 | 513 | – | 519 | 410.7620 | 819.5094 | 819.4854 | 0.0240 | 0 | 12 | 3.4 | 10Score **> 43** indicates **identity** Score **> 29** indicates **homology** | U | K.IVGLQYK.K |
| 127 | 513 | – | 520 | 474.8290 | 947.6434 | 947.5804 | 0.0631 | 1 | 28 | 0.19 | 1Score **> 43** indicates **identity** Score **> 33** indicates **homology** | U | K.IVGLQYKK.N |
| 166 | 521 | – | 529 | 556.7600 | 1111.5054 | 1111.4669 | 0.0385 | 0 | 47 | 0.016 | 1Score **> 42** indicates **identity** | U | K.NYEDEDSLK.T |
| 236 | 536 | – | 550 | 588.6050 | 1762.7932 | 1762.7866 | 0.0066 | 0 | 25 | 0.21 | 1Score **> 41** indicates **identity** Score **> 30** indicates **homology** | U | K.IMIMTDQDQDGSHIK.G + 2 Oxidation (M) |
| 203 | 569 | – | 579 | 668.3730 | 1334.7314 | 1334.7486 | -0.0171 | 0 | 43 | 0.0035 | 1Score **> 42** indicates **identity** Score **> 31** indicates **homology** | U | R.FLEEFITPIVK.V |
| 153 | 626 | – | 633 | 538.2960 | 1074.5774 | 1074.4804 | 0.0970 | 1 | 22 | 0.14 | 1Score **> 43** indicates **identity** Score **> 26** indicates **homology** | U | K.EYFADMKR.H + Oxidation (M) |
| 232 | 640 | – | 655 | 835.8590 | 1669.7034 | 1669.7835 | -0.0801 | 0 | 89 | 4.6e-08 | 1Score **> 41** indicates **identity** Score **> 28** indicates **homology** | U | K.YSGPEDDAAISLAFSK.K |
| 216 | 714 | – | 727 | 750.8820 | 1499.7494 | 1499.7766 | -0.0272 | 0 | 36 | 0.0019 | 1Score **> 42** indicates **identity** Score **> 22** indicates **homology** | U | R.SIPSMVDGLKPGQR.K + Oxidation (M) |
| 201 | 805 | – | 815 | 659.3380 | 1316.6614 | 1316.6798 | -0.0184 | 0 | 74 | 4e-05 | 1Score **> 42** indicates **identity** | U | R.YIFTMLSSLAR.L + Oxidation (M) |
| 106 | 871 | – | 877 | 429.2650 | 856.5154 | 856.4766 | 0.0388 | 0 | 36 | 0.3 | 1Score **> 43** indicates **identity** | U | R.EIVNNIR.R |
| 140 | 871 | – | 878 | 507.3070 | 1012.5994 | 1012.5777 | 0.0217 | 1 | 15 | 4.6 | 8Score **> 42** indicates **identity** Score **> 34** indicates **homology** | U | R.EIVNNIRR.L |
| 124 | 930 | – | 936 | 464.2560 | 926.4974 | 926.4498 | 0.0477 | 0 | 23 | 0.75 | 1Score **> 42** indicates **identity** Score **> 35** indicates **homology** | U | R.TWTQTYK.E |
| 154 | 950 | – | 958 | 538.3130 | 1074.6114 | 1074.5710 | 0.0405 | 0 | 47 | 0.013 | 1Score **> 43** indicates **identity** Score **> 41** indicates **homology** | U | K.TPPLITDYR.E |
| 162 | 959 | – | 967 | 547.2760 | 1092.5374 | 1092.5088 | 0.0287 | 0 | 32 | 0.086 | 1Score **> 42** indicates **identity** Score **> 34** indicates **homology** | U | R.EYHTDTTVK.F |
| 129 | 1063 | – | 1070 | 477.8350 | 953.6554 | 953.5909 | 0.0645 | 0 | 39 | 0.093 | 1Score **> 42** indicates **identity** | U | K.IIIENKPK.K |
| 172 | 1152 | – | 1160 | 566.3010 | 1130.5874 | 1130.5931 | -0.0057 | 1 | 26 | 0.12 | 1Score **> 43** indicates **identity** Score **> 29** indicates **homology** | U | K.EQELDTLKR.K |
| 44 | 1205 | – | 1215 | 614.8490 | 1227.6834 | 1227.6281 | 0.0553 | 0 | 34 | 0.0094 | 1Score **> 43** indicates **identity** Score **> 26** indicates **homology** | U | K.TQMAEVLPSPR.G |
| 193 | 1205 | – | 1215 | 622.8020 | 1243.5894 | 1243.6231 | -0.0336 | 0 | 46 | 0.0068 | 1Score **> 42** indicates **identity** Score **> 36** indicates **homology** | U | K.TQMAEVLPSPR.G + Oxidation (M) |

---

```
AC   Q9UQP9;
DT   01-JUL-1989, integrated into UniProtKB/Swiss-Prot.
DT   04-MAY-2001, sequence version 3.
DT   29-OCT-2014, entry version 185.
DE   RecName: Full=DNA topoisomerase 2-alpha;
DE            EC=5.99.1.3;
DE   AltName: Full=DNA topoisomerase II, alpha isozyme;
GN   Name=TOP2A; Synonyms=TOP2;
OS   Homo sapiens (Human).
OC   Eukaryota; Metazoa; Chordata; Craniata; Vertebrata; Euteleostomi;
OC   Mammalia; Eutheria; Euarchontoglires; Primates; Haplorrhini;
OC   Catarrhini; Hominidae; Homo.
OX   NCBI_TaxID=9606;
RN   [1]
RP   NUCLEOTIDE SEQUENCE [MRNA] (ISOFORM 1).
RX   PubMed=2845399; DOI=10.1073/pnas.85.19.7177;
RA   Tsai-Pflugfelder M., Liu L.F., Liu A.A., Tewey K.M., Whang-Peng J.,
RA   Knutsen T., Huebner K., Croce C.M., Wang J.C.;
RT   "Cloning and sequencing of cDNA encoding human DNA topoisomerase II
RT   and localization of the gene to chromosome region 17q21-22.";
RL   Proc. Natl. Acad. Sci. U.S.A. 85:7177-7181(1988).
RN   [2]
RP   SEQUENCE REVISION TO 109-114.
RX   PubMed=8393377;
RA   Wasserman R.A., Austin C.A., Fisher L.M., Wang J.C.;
RT   "Use of yeast in the study of anticancer drugs targeting DNA
RT   topoisomerases: expression of a functional recombinant human DNA
RT   topoisomerase II alpha in yeast.";
RL   Cancer Res. 53:3591-3596(1993).
RN   [3]
RP   NUCLEOTIDE SEQUENCE [GENOMIC DNA] (ISOFORM 1).
RX   PubMed=9795238; DOI=10.1016/S0378-1119(98)00468-5;
RA   Lang A.J., Mirski S.E., Cummings H.J., Yu Q., Gerlach J.H., Cole S.P.;
RT   "Structural organization of the human TOP2A and TOP2B genes.";
RL   Gene 221:255-266(1998).
RN   [4]
RP   NUCLEOTIDE SEQUENCE [GENOMIC DNA] (ISOFORM 1).
RX   PubMed=10095062; DOI=10.1016/S0167-4781(99)00020-2;
RA   Sng J.H., Heaton V.J., Bell M., Mani P., Austin C.A., Fisher L.M.;
RT   "Molecular cloning and characterization of the human topoisomerase
RT   IIalpha and IIbeta genes: evidence for isoform evolution through gene
RT   duplication.";
RL   Biochim. Biophys. Acta 1444:395-406(1999).
RN   [5]
RP   NUCLEOTIDE SEQUENCE [LARGE SCALE GENOMIC DNA].
RX   PubMed=16625196; DOI=10.1038/nature04689;
RA   Zody M.C., Garber M., Adams D.J., Sharpe T., Harrow J., Lupski J.R.,
RA   Nicholson C., Searle S.M., Wilming L., Young S.K., Abouelleil A.,
RA   Allen N.R., Bi W., Bloom T., Borowsky M.L., Bugalter B.E., Butler J.,
RA   Chang J.L., Chen C.-K., Cook A., Corum B., Cuomo C.A., de Jong P.J.,
RA   DeCaprio D., Dewar K., FitzGerald M., Gilbert J., Gibson R.,
RA   Gnerre S., Goldstein S., Grafham D.V., Grocock R., Hafez N.,
RA   Hagopian D.S., Hart E., Norman C.H., Humphray S., Jaffe D.B.,
RA   Jones M., Kamal M., Khodiyar V.K., LaButti K., Laird G., Lehoczky J.,
RA   Liu X., Lokyitsang T., Loveland J., Lui A., Macdonald P., Major J.E.,
RA   Matthews L., Mauceli E., McCarroll S.A., Mihalev A.H., Mudge J.,
RA   Nguyen C., Nicol R., O'Leary S.B., Osoegawa K., Schwartz D.C.,
RA   Shaw-Smith C., Stankiewicz P., Steward C., Swarbreck D.,
RA   Venkataraman V., Whittaker C.A., Yang X., Zimmer A.R., Bradley A.,
RA   Hubbard T., Birren B.W., Rogers J., Lander E.S., Nusbaum C.;
RT   "DNA sequence of human chromosome 17 and analysis of rearrangement in
RT   the human lineage.";
RL   Nature 440:1045-1049(2006).
RN   [6]
RP   NUCLEOTIDE SEQUENCE [LARGE SCALE GENOMIC DNA].
RA   Mural R.J., Istrail S., Sutton G.G., Florea L., Halpern A.L.,
RA   Mobarry C.M., Lippert R., Walenz B., Shatkay H., Dew I., Miller J.R.,
RA   Flanigan M.J., Edwards N.J., Bolanos R., Fasulo D., Halldorsson B.V.,
RA   Hannenhalli S., Turner R., Yooseph S., Lu F., Nusskern D.R.,
RA   Shue B.C., Zheng X.H., Zhong F., Delcher A.L., Huson D.H.,
RA   Kravitz S.A., Mouchard L., Reinert K., Remington K.A., Clark A.G.,
RA   Waterman M.S., Eichler E.E., Adams M.D., Hunkapiller M.W., Myers E.W.,
RA   Venter J.C.;
RL   Submitted (JUL-2005) to the EMBL/GenBank/DDBJ databases.
RN   [7]
RP   NUCLEOTIDE SEQUENCE [LARGE SCALE MRNA] (ISOFORM 1).
RX   PubMed=15489334; DOI=10.1101/gr.2596504;
RG   The MGC Project Team;
RT   "The status, quality, and expansion of the NIH full-length cDNA
RT   project: the Mammalian Gene Collection (MGC).";
RL   Genome Res. 14:2121-2127(2004).
RN   [8]
RP   NUCLEOTIDE SEQUENCE [MRNA] OF 1-500 (ISOFORMS 2; 3 AND 4).
RA   Petruti-Mot A.S., Earnshaw W.C.;
RT   "Two differentially spliced forms of topoisomerase II alpha and beta
RT   mRNAs are conserved between birds and humans.";
RL   Submitted (JUL-2000) to the EMBL/GenBank/DDBJ databases.
RN   [9]
RP   NUCLEOTIDE SEQUENCE [GENOMIC DNA] OF 1-21 AND 48-72.
RA   Neri S., Govoni M., Perrotta L., Pozzi S., Pession A.;
RL   Submitted (JUN-1998) to the EMBL/GenBank/DDBJ databases.
RN   [10]
RP   PROTEIN SEQUENCE OF 1-17; 74-96; 124-131; 169-184; 243-251; 277-287;
RP   325-336; 387-397; 467-478; 481-487; 500-519; 569-579; 702-713;
RP   805-815; 828-835; 864-877; 937-958; 1021-1026 AND 1185-1196,
RP   ACETYLATION AT MET-1, AND IDENTIFICATION BY MASS SPECTROMETRY.
RC   TISSUE=Ovarian carcinoma;
RA   Bienvenut W.V., Lilla S., von Kriegsheim A., Lempens A., Kolch W.;
RL   Submitted (DEC-2008) to UniProtKB.
RN   [11]
RP   PROTEIN SEQUENCE OF 135-157; 228-241; 307-321; 325-336; 387-397;
RP   401-416; 467-478; 536-550; 569-579; 640-655; 702-713; 805-815;
RP   1011-1020; 1097-1114; 1169-1184; 1239-1259 AND 1374-1411,
RP   PHOSPHORYLATION AT SER-1106, AND IDENTIFICATION BY MASS SPECTROMETRY.
RC   TISSUE=Cervix carcinoma;
RA   Bienvenut W.V., Waridel P., Quadroni M.;
RL   Submitted (MAR-2009) to UniProtKB.
RN   [12]
RP   PHOSPHORYLATION AT SER-1469, AND MUTAGENESIS OF SER-1469.
RX   PubMed=10942766; DOI=10.1074/jbc.M005179200;
RA   Escargueil A.E., Plisov S.Y., Filhol O., Cochet C., Larsen A.K.;
RT   "Mitotic phosphorylation of DNA topoisomerase II alpha by protein
RT   kinase CK2 creates the MPM-2 phosphoepitope on Ser-1469.";
RL   J. Biol. Chem. 275:34710-34718(2000).
RN   [13]
RP   NUCLEAR EXPORT SIGNAL.
RX   PubMed=12821127; DOI=10.1016/S0006-291X(03)01077-5;
RA   Mirski S.E., Bielawski J.C., Cole S.P.;
RT   "Identification of functional nuclear export sequences in human
RT   topoisomerase IIalpha and beta.";
RL   Biochem. Biophys. Res. Commun. 306:905-911(2003).
RN   [14]
RP   INTERACTION WITH COPS5.
RX   PubMed=15126503; DOI=10.1074/jbc.M401411200;
RA   Yun J., Tomida A., Andoh T., Tsuruo T.;
RT   "Interaction between glucose-regulated destruction domain of DNA
RT   topoisomerase IIalpha and MPN domain of Jab1/CSN5.";
RL   J. Biol. Chem. 279:31296-31303(2004).
RN   [15]
RP   PHOSPHORYLATION [LARGE SCALE ANALYSIS] AT SER-4; SER-1106; SER-1213;
RP   SER-1247; SER-1332; SER-1337; THR-1343; SER-1351; SER-1354; SER-1374;
RP   SER-1377 AND SER-1525, AND IDENTIFICATION BY MASS SPECTROMETRY [LARGE
RP   SCALE ANALYSIS].
RC   TISSUE=Cervix carcinoma;
RX   PubMed=17081983; DOI=10.1016/j.cell.2006.09.026;
RA   Olsen J.V., Blagoev B., Gnad F., Macek B., Kumar C., Mortensen P.,
RA   Mann M.;
RT   "Global, in vivo, and site-specific phosphorylation dynamics in
RT   signaling networks.";
RL   Cell 127:635-648(2006).
RN   [16]
RP   PHOSPHORYLATION [LARGE SCALE ANALYSIS] AT SER-1213; SER-1374; SER-1377
RP   AND SER-1525, AND IDENTIFICATION BY MASS SPECTROMETRY [LARGE SCALE
RP   ANALYSIS].
RC   TISSUE=Cervix carcinoma;
RX   PubMed=16964243; DOI=10.1038/nbt1240;
RA   Beausoleil S.A., Villen J., Gerber S.A., Rush J., Gygi S.P.;
RT   "A probability-based approach for high-throughput protein
RT   phosphorylation analysis and site localization.";
RL   Nat. Biotechnol. 24:1285-1292(2006).
RN   [17]
RP   PHOSPHORYLATION [LARGE SCALE ANALYSIS] AT SER-1247, AND IDENTIFICATION
RP   BY MASS SPECTROMETRY [LARGE SCALE ANALYSIS].
RC   TISSUE=Cervix carcinoma;
RX   PubMed=17924679; DOI=10.1021/pr070152u;
RA   Yu L.R., Zhu Z., Chan K.C., Issaq H.J., Dimitrov D.S., Veenstra T.D.;
RT   "Improved titanium dioxide enrichment of phosphopeptides from HeLa
RT   cells and high confident phosphopeptide identification by cross-
RT   validation of MS/MS and MS/MS/MS spectra.";
RL   J. Proteome Res. 6:4150-4162(2007).
RN   [18]
RP   PHOSPHORYLATION AT THR-1343.
RX   PubMed=18062778; DOI=10.1042/BJ20071394;
RA   Iida M., Matsuda M., Komatani H.;
RT   "Plk3 phosphorylates topoisomerase IIalpha at Thr(1342), a site that
RT   is not recognized by Plk1.";
RL   Biochem. J. 411:27-32(2008).
RN   [19]
RP   PHOSPHORYLATION [LARGE SCALE ANALYSIS] AT SER-1504, AND IDENTIFICATION
RP   BY MASS SPECTROMETRY [LARGE SCALE ANALYSIS].
RC   TISSUE=Cervix carcinoma;
RX   PubMed=18220336; DOI=10.1021/pr0705441;
RA   Cantin G.T., Yi W., Lu B., Park S.K., Xu T., Lee J.-D.,
RA   Yates J.R. III;
RT   "Combining protein-based IMAC, peptide-based IMAC, and MudPIT for
RT   efficient phosphoproteomic analysis.";
RL   J. Proteome Res. 7:1346-1351(2008).
RN   [20]
RP   PHOSPHORYLATION [LARGE SCALE ANALYSIS] AT SER-4; SER-1213; SER-1247;
RP   SER-1332; SER-1337 AND SER-1377, AND IDENTIFICATION BY MASS
RP   SPECTROMETRY [LARGE SCALE ANALYSIS].
RC   TISSUE=Cervix carcinoma;
RX   PubMed=18691976; DOI=10.1016/j.molcel.2008.07.007;
RA   Daub H., Olsen J.V., Bairlein M., Gnad F., Oppermann F.S., Korner R.,
RA   Greff Z., Keri G., Stemmann O., Mann M.;
RT   "Kinase-selective enrichment enables quantitative phosphoproteomics of
RT   the kinome across the cell cycle.";
RL   Mol. Cell 31:438-448(2008).
RN   [21]
RP   PHOSPHORYLATION [LARGE SCALE ANALYSIS] AT SER-1106; SER-1213;
RP   SER-1332; SER-1337; THR-1343; SER-1351; SER-1392; SER-1393; SER-1449;
RP   SER-1469; THR-1470; SER-1471; SER-1474 AND SER-1525, AND
RP   IDENTIFICATION BY MASS SPECTROMETRY [LARGE SCALE ANALYSIS].
RC   TISSUE=Cervix carcinoma;
RX   PubMed=18669648; DOI=10.1073/pnas.0805139105;
RA   Dephoure N., Zhou C., Villen J., Beausoleil S.A., Bakalarski C.E.,
RA   Elledge S.J., Gygi S.P.;
RT   "A quantitative atlas of mitotic phosphorylation.";
RL   Proc. Natl. Acad. Sci. U.S.A. 105:10762-10767(2008).
RN   [22]
RP   IDENTIFICATION BY MASS SPECTROMETRY [LARGE SCALE ANALYSIS].
RX   PubMed=19413330; DOI=10.1021/ac9004309;
RA   Gauci S., Helbig A.O., Slijper M., Krijgsveld J., Heck A.J.,
RA   Mohammed S.;
RT   "Lys-N and trypsin cover complementary parts of the phosphoproteome in
RT   a refined SCX-based approach.";
RL   Anal. Chem. 81:4493-4501(2009).
RN   [23]
RP   CATALYTIC ACTIVITY, AND COFACTOR.
RX   PubMed=19222228; DOI=10.1021/bi8023256;
RA   Deweese J.E., Burch A.M., Burgin A.B., Osheroff N.;
RT   "Use of divalent metal ions in the DNA cleavage reaction of human type
RT   II topoisomerases.";
RL   Biochemistry 48:1862-1869(2009).
RN   [24]
RP   MUTAGENESIS OF GLU-461; ASP-541; ASP-543 AND ASP-545, PUTATIVE
RP   METAL-BINDING SITES, CATALYTIC ACTIVITY, AND COFACTOR.
RX   PubMed=19697956; DOI=10.1021/bi900875c;
RA   Deweese J.E., Guengerich F.P., Burgin A.B., Osheroff N.;
RT   "Metal ion interactions in the DNA cleavage/ligation active site of
RT   human topoisomerase IIalpha.";
RL   Biochemistry 48:8940-8947(2009).
RN   [25]
RP   PHOSPHORYLATION AT SER-1106 BY CSNK1D/CK1.
RX   PubMed=19043076; DOI=10.1093/nar/gkn934;
RA   Grozav A.G., Chikamori K., Kozuki T., Grabowski D.R., Bukowski R.M.,
RA   Willard B., Kinter M., Andersen A.H., Ganapathi R., Ganapathi M.K.;
RT   "Casein kinase I delta/epsilon phosphorylates topoisomerase IIalpha at
RT   serine-1106 and modulates DNA cleavage activity.";
RL   Nucleic Acids Res. 37:382-392(2009).
RN   [26]
RP   PHOSPHORYLATION [LARGE SCALE ANALYSIS] AT SER-1247; SER-1374;
RP   SER-1387; SER-1393; SER-1504 AND SER-1525, AND IDENTIFICATION BY MASS
RP   SPECTROMETRY [LARGE SCALE ANALYSIS].
RC   TISSUE=Leukemic T-cell;
RX   PubMed=19690332; DOI=10.1126/scisignal.2000007;
RA   Mayya V., Lundgren D.H., Hwang S.-I., Rezaul K., Wu L., Eng J.K.,
RA   Rodionov V., Han D.K.;
RT   "Quantitative phosphoproteomic analysis of T cell receptor signaling
RT   reveals system-wide modulation of protein-protein interactions.";
RL   Sci. Signal. 2:RA46-RA46(2009).
RN   [27]
RP   ACETYLATION [LARGE SCALE ANALYSIS] AT MET-1, PHOSPHORYLATION [LARGE
RP   SCALE ANALYSIS] AT SER-4; THR-282; SER-1106; THR-1205; SER-1213;
RP   SER-1247; SER-1332; SER-1337; THR-1343; SER-1351; SER-1354; SER-1374;
RP   SER-1377; SER-1471; SER-1474; SER-1495; SER-1504 AND SER-1525, AND
RP   IDENTIFICATION BY MASS SPECTROMETRY [LARGE SCALE ANALYSIS].
RC   TISSUE=Cervix carcinoma;
RX   PubMed=20068231; DOI=10.1126/scisignal.2000475;
RA   Olsen J.V., Vermeulen M., Santamaria A., Kumar C., Miller M.L.,
RA   Jensen L.J., Gnad F., Cox J., Jensen T.S., Nigg E.A., Brunak S.,
RA   Mann M.;
RT   "Quantitative phosphoproteomics reveals widespread full
RT   phosphorylation site occupancy during mitosis.";
RL   Sci. Signal. 3:RA3-RA3(2010).
RN   [28]
RP   IDENTIFICATION BY MASS SPECTROMETRY [LARGE SCALE ANALYSIS].
RX   PubMed=21269460; DOI=10.1186/1752-0509-5-17;
RA   Burkard T.R., Planyavsky M., Kaupe I., Breitwieser F.P.,
RA   Buerckstuemmer T., Bennett K.L., Superti-Furga G., Colinge J.;
RT   "Initial characterization of the human central proteome.";
RL   BMC Syst. Biol. 5:17-17(2011).
RN   [29]
RP   ACETYLATION [LARGE SCALE ANALYSIS] AT MET-1, PHOSPHORYLATION [LARGE
RP   SCALE ANALYSIS] AT SER-4; SER-1106; SER-1213; SER-1247; SER-1295;
RP   SER-1297; SER-1299; SER-1302; SER-1332; SER-1337; THR-1343; SER-1351;
RP   SER-1354; SER-1374; SER-1377; SER-1469; THR-1470; SER-1471; SER-1474;
RP   SER-1476 AND SER-1525, AND IDENTIFICATION BY MASS SPECTROMETRY [LARGE
RP   SCALE ANALYSIS].
RX   PubMed=21406692; DOI=10.1126/scisignal.2001570;
RA   Rigbolt K.T., Prokhorova T.A., Akimov V., Henningsen J.,
RA   Johansen P.T., Kratchmarova I., Kassem M., Mann M., Olsen J.V.,
RA   Blagoev B.;
RT   "System-wide temporal characterization of the proteome and
RT   phosphoproteome of human embryonic stem cell differentiation.";
RL   Sci. Signal. 4:RS3-RS3(2011).
RN   [30]
RP   MUTAGENESIS OF 342-LYS--LYS-344.
RX   PubMed=23022727; DOI=10.1038/nsmb.2388;
RA   Schmidt B.H., Osheroff N., Berger J.M.;
RT   "Structure of a topoisomerase II-DNA-nucleotide complex reveals a new
RT   control mechanism for ATPase activity.";
RL   Nat. Struct. Mol. Biol. 19:1147-1154(2012).
RN   [31]
RP   FUNCTION, SUBCELLULAR LOCATION, AND INTERACTION WITH RECQL5.
RX   PubMed=22013166; DOI=10.1093/nar/gkr844;
RA   Ramamoorthy M., Tadokoro T., Rybanska I., Ghosh A.K., Wersto R.,
RA   May A., Kulikowicz T., Sykora P., Croteau D.L., Bohr V.A.;
RT   "RECQL5 cooperates with Topoisomerase II alpha in DNA decatenation and
RT   cell cycle progression.";
RL   Nucleic Acids Res. 40:1621-1635(2012).
RN   [32]
RP   ACETYLATION [LARGE SCALE ANALYSIS] AT MET-1, AND IDENTIFICATION BY
RP   MASS SPECTROMETRY [LARGE SCALE ANALYSIS].
RX   PubMed=22814378; DOI=10.1073/pnas.1210303109;
RA   Van Damme P., Lasa M., Polevoda B., Gazquez C., Elosegui-Artola A.,
RA   Kim D.S., De Juan-Pardo E., Demeyer K., Hole K., Larrea E.,
RA   Timmerman E., Prieto J., Arnesen T., Sherman F., Gevaert K.,
RA   Aldabe R.;
RT   "N-terminal acetylome analyses and functional insights of the N-
RT   terminal acetyltransferase NatB.";
RL   Proc. Natl. Acad. Sci. U.S.A. 109:12449-12454(2012).
RN   [33]
RP   FUNCTION, MUTAGENESIS OF GLU-461; ASP-541 AND ASP-543, AND COFACTOR.
RX   PubMed=22323612; DOI=10.1073/pnas.1115704109;
RA   Lee S., Jung S.R., Heo K., Byl J.A., Deweese J.E., Osheroff N.,
RA   Hohng S.;
RT   "DNA cleavage and opening reactions of human topoisomerase IIalpha are
RT   regulated via Mg2+-mediated dynamic bending of gate-DNA.";
RL   Proc. Natl. Acad. Sci. U.S.A. 109:2925-2930(2012).
RN   [34]
RP   X-RAY CRYSTALLOGRAPHY (2.51 ANGSTROMS) OF 29-428 IN COMPLEX WITH ADP,
RP   AND X-RAY CRYSTALLOGRAPHY (1.87 ANGSTROMS) OF 29-428 IN COMPLEX WITH
RP   AMPPNP.
RX   PubMed=16100112; DOI=10.1074/jbc.M506520200;
RA   Wei H., Ruthenburg A.J., Bechis S.K., Verdine G.L.;
RT   "Nucleotide-dependent domain movement in the ATPase domain of a human
RT   type IIA DNA topoisomerase.";
RL   J. Biol. Chem. 280:37041-37047(2005).
RN   [35]
RP   X-RAY CRYSTALLOGRAPHY (2.9 ANGSTROMS) OF 431-1193 IN COMPLEX WITH DNA
RP   AND MAGNESIUM ION, SUBUNIT, AND COFACTOR.
RX   PubMed=22841979; DOI=10.1016/j.jmb.2012.07.014;
RA   Wendorff T.J., Schmidt B.H., Heslop P., Austin C.A., Berger J.M.;
RT   "The structure of DNA-bound human topoisomerase II alpha:
RT   conformational mechanisms for coordinating inter-subunit interactions
RT   with DNA cleavage.";
RL   J. Mol. Biol. 424:109-124(2012).
RN   [36]
RP   VARIANT AMSACRINE-RESISTANT LYS-487.
RX   PubMed=1651812;
RA   Hinds M., Deisseroth K., Mayes J., Altschuler E., Jansen R.,
RA   Ledley F.D., Zwelling L.A.;
RT   "Identification of a point mutation in the topoisomerase II gene from
RT   a human leukemia cell line containing an amsacrine-resistant form of
RT   topoisomerase II.";
RL   Cancer Res. 51:4729-4731(1991).
RN   [37]
RP   VARIANT TENIPOSIDE-RESISTANT GLN-450.
RX   PubMed=1652758; DOI=10.1073/pnas.88.17.7654;
RA   Bugg B.Y., Danks M.K., Beck W.T., Suttle D.P.;
RT   "Expression of a mutant DNA topoisomerase II in CCRF-CEM human
RT   leukemic cells selected for resistance to teniposide.";
RL   Proc. Natl. Acad. Sci. U.S.A. 88:7654-7658(1991).
CC   -!- FUNCTION: Control of topological states of DNA by transient
CC       breakage and subsequent rejoining of DNA strands. Topoisomerase II
CC       makes double-strand breaks. Essential during mitosis and meiosis
CC       for proper segregation of daughter chromosomes.
CC       {ECO:0000269|PubMed:22013166, ECO:0000269|PubMed:22323612}.
CC   -!- CATALYTIC ACTIVITY: ATP-dependent breakage, passage and rejoining
CC       of double-stranded DNA. {ECO:0000255|PROSITE-ProRule:PRU00995,
CC       ECO:0000269|PubMed:19222228, ECO:0000269|PubMed:19697956}.
CC   -!- COFACTOR: Magnesium. Binds two Mg(2+) per subunit. The magnesium
CC       ions form salt bridges with both the protein and the DNA. Can also
CC       accept other divalent metal cations, such as Mn(2+) and Ca(2+)
CC       (Probable). {ECO:0000305|PubMed:19222228,
CC       ECO:0000305|PubMed:19697956, ECO:0000305|PubMed:22323612,
CC       ECO:0000305|PubMed:22841979}.
CC   -!- ENZYME REGULATION: Specifically inhibited by the intercalating
CC       agent amsacrine.
CC   -!- SUBUNIT: Homodimer. Interacts with COPS5. Interacts with RECQL5;
CC       this stimulates DNA decatenation. {ECO:0000269|PubMed:15126503,
CC       ECO:0000269|PubMed:16100112, ECO:0000269|PubMed:22013166,
CC       ECO:0000269|PubMed:22841979}.
CC   -!- INTERACTION:
CC       P35222:CTNNB1; NbExp=5; IntAct=EBI-539628, EBI-491549;
CC   -!- SUBCELLULAR LOCATION: Cytoplasm {ECO:0000269|PubMed:22013166}.
CC       Nucleus, nucleoplasm {ECO:0000269|PubMed:22013166}. Note=Generally
CC       located in the nucleoplasm.
CC   -!- ALTERNATIVE PRODUCTS:
CC       Event=Alternative splicing; Named isoforms=4;
CC       Name=1;
CC         IsoId=P11388-1; Sequence=Displayed;
CC       Name=2;
CC         IsoId=P11388-2; Sequence=VSP_006531;
CC       Name=3;
CC         IsoId=P11388-3; Sequence=VSP_006529;
CC       Name=4;
CC         IsoId=P11388-4; Sequence=VSP_006530;
CC   -!- PTM: Phosphorylation has no effect on catalytic activity. However,
CC       phosphorylation at Ser-1106 by CSNK1D/CK1 promotes DNA cleavable
CC       complex formation. {ECO:0000269|PubMed:10942766,
CC       ECO:0000269|PubMed:16964243, ECO:0000269|PubMed:17081983,
CC       ECO:0000269|PubMed:17924679, ECO:0000269|PubMed:18062778,
CC       ECO:0000269|PubMed:18220336, ECO:0000269|PubMed:18669648,
CC       ECO:0000269|PubMed:18691976, ECO:0000269|PubMed:19043076,
CC       ECO:0000269|PubMed:19690332, ECO:0000269|PubMed:20068231,
CC       ECO:0000269|PubMed:21406692, ECO:0000269|Ref.11}.
CC   -!- MISCELLANEOUS: Eukaryotic topoisomerase I and II can relax both
CC       negative and positive supercoils, whereas prokaryotic enzymes
CC       relax only negative supercoils.
CC   -!- SIMILARITY: Belongs to the type II topoisomerase family.
CC       {ECO:0000305}.
CC   -!- SIMILARITY: Contains 1 Toprim domain. {ECO:0000255|PROSITE-
CC       ProRule:PRU00995}.
CC   -----------------------------------------------------------------------
CC   Copyrighted by the UniProt Consortium, see http://www.uniprot.org/terms
CC   Distributed under the Creative Commons Attribution-NoDerivs License
CC   -----------------------------------------------------------------------
DR   EMBL; J04088; AAA61209.1; -; mRNA.
DR   EMBL; AF071747; AAC77388.1; -; Genomic_DNA.
DR   EMBL; AF071738; AAC77388.1; JOINED; Genomic_DNA.
DR   EMBL; AF071739; AAC77388.1; JOINED; Genomic_DNA.
DR   EMBL; AF071740; AAC77388.1; JOINED; Genomic_DNA.
DR   EMBL; AF071741; AAC77388.1; JOINED; Genomic_DNA.
DR   EMBL; AF071742; AAC77388.1; JOINED; Genomic_DNA.
DR   EMBL; AF071743; AAC77388.1; JOINED; Genomic_DNA.
DR   EMBL; AF071744; AAC77388.1; JOINED; Genomic_DNA.
DR   EMBL; AF071745; AAC77388.1; JOINED; Genomic_DNA.
DR   EMBL; AF071746; AAC77388.1; JOINED; Genomic_DNA.
DR   EMBL; AJ011741; CAA09762.1; -; Genomic_DNA.
DR   EMBL; AJ011742; CAA09762.1; JOINED; Genomic_DNA.
DR   EMBL; AJ011743; CAA09762.1; JOINED; Genomic_DNA.
DR   EMBL; AJ011744; CAA09762.1; JOINED; Genomic_DNA.
DR   EMBL; AJ011745; CAA09762.1; JOINED; Genomic_DNA.
DR   EMBL; AJ011746; CAA09762.1; JOINED; Genomic_DNA.
DR   EMBL; AJ011747; CAA09762.1; JOINED; Genomic_DNA.
DR   EMBL; AJ011748; CAA09762.1; JOINED; Genomic_DNA.
DR   EMBL; AJ011749; CAA09762.1; JOINED; Genomic_DNA.
DR   EMBL; AJ011750; CAA09762.1; JOINED; Genomic_DNA.
DR   EMBL; AJ011751; CAA09762.1; JOINED; Genomic_DNA.
DR   EMBL; AJ011752; CAA09762.1; JOINED; Genomic_DNA.
DR   EMBL; AJ011753; CAA09762.1; JOINED; Genomic_DNA.
DR   EMBL; AJ011754; CAA09762.1; JOINED; Genomic_DNA.
DR   EMBL; AJ011755; CAA09762.1; JOINED; Genomic_DNA.
DR   EMBL; AJ011756; CAA09762.1; JOINED; Genomic_DNA.
DR   EMBL; AJ011757; CAA09762.1; JOINED; Genomic_DNA.
DR   EMBL; AJ011758; CAA09762.1; JOINED; Genomic_DNA.
DR   EMBL; AC080112; -; NOT_ANNOTATED_CDS; Genomic_DNA.
DR   EMBL; AF285157; AAG13403.1; -; mRNA.
DR   EMBL; AF285158; AAG13404.1; -; mRNA.
DR   EMBL; CH471152; EAW60663.1; -; Genomic_DNA.
DR   EMBL; BC140791; AAI40792.1; -; mRNA.
DR   EMBL; AF285159; AAG13405.1; -; mRNA.
DR   EMBL; AF069522; AAC23518.1; -; Genomic_DNA.
DR   EMBL; AF064590; AAC16736.1; -; Genomic_DNA.
DR   CCDS; CCDS45672.1; -. [P11388-1]
DR   PIR; A40493; A40493.
DR   RefSeq; NP_001058.2; NM_001067.3. [P11388-1]
DR   UniGene; Hs.156346; -.
DR   PDB; 1LWZ; Model; -; A=431-1200.
DR   PDB; 1ZXM; X-ray; 1.87 A; A/B=29-428.
DR   PDB; 1ZXN; X-ray; 2.51 A; A/B/C/D=29-428.
DR   PDB; 4FM9; X-ray; 2.90 A; A=431-1193.
DR   PDBsum; 1LWZ; -.
DR   PDBsum; 1ZXM; -.
DR   PDBsum; 1ZXN; -.
DR   PDBsum; 4FM9; -.
DR   ProteinModelPortal; P11388; -.
DR   SMR; P11388; 29-1190.
DR   BioGrid; 113006; 62.
DR   DIP; DIP-33887N; -.
DR   IntAct; P11388; 17.
DR   MINT; MINT-98770; -.
DR   BindingDB; P11388; -.
DR   ChEMBL; CHEMBL1806; -.
DR   DrugBank; DB00276; Amsacrine.
DR   DrugBank; DB00537; Ciprofloxacin.
DR   DrugBank; DB00970; Dactinomycin.
DR   DrugBank; DB00694; Daunorubicin.
DR   DrugBank; DB00380; Dexrazoxane.
DR   DrugBank; DB00997; Doxorubicin.
DR   DrugBank; DB00467; Enoxacin.
DR   DrugBank; DB00445; Epirubicin.
DR   DrugBank; DB00773; Etoposide.
DR   DrugBank; DB04576; Fleroxacin.
DR   DrugBank; DB01177; Idarubicin.
DR   DrugBank; DB01137; Levofloxacin.
DR   DrugBank; DB00978; Lomefloxacin.
DR   DrugBank; DB04967; Lucanthone.
DR   DrugBank; DB01204; Mitoxantrone.
DR   DrugBank; DB00218; Moxifloxacin.
DR   DrugBank; DB01059; Norfloxacin.
DR   DrugBank; DB01165; Ofloxacin.
DR   DrugBank; DB00487; Pefloxacin.
DR   DrugBank; DB01179; Podofilox.
DR   DrugBank; DB01208; Sparfloxacin.
DR   DrugBank; DB00444; Teniposide.
DR   DrugBank; DB00685; Trovafloxacin.
DR   DrugBank; DB00385; Valrubicin.
DR   GuidetoPHARMACOLOGY; 2637; -.
DR   PhosphoSite; P11388; -.
DR   DMDM; 13959709; -.
DR   UCD-2DPAGE; P11388; -.
DR   MaxQB; P11388; -.
DR   PaxDb; P11388; -.
DR   PRIDE; P11388; -.
DR   Ensembl; ENST00000423485; ENSP00000411532; ENSG00000131747. [P11388-1]
DR   GeneID; 7153; -.
DR   KEGG; hsa:7153; -.
DR   UCSC; uc002huq.3; human. [P11388-1]
DR   CTD; 7153; -.
DR   GeneCards; GC17M038544; -.
DR   H-InvDB; HIX0013797; -.
DR   HGNC; HGNC:11989; TOP2A.
DR   HPA; CAB002448; -.
DR   HPA; HPA006458; -.
DR   HPA; HPA026773; -.
DR   MIM; 126430; gene.
DR   neXtProt; NX_P11388; -.
DR   Orphanet; 635; Neuroblastoma.
DR   PharmGKB; PA354; -.
DR   eggNOG; COG0187; -.
DR   GeneTree; ENSGT00390000016222; -.
DR   HOVERGEN; HBG052998; -.
DR   InParanoid; P11388; -.
DR   KO; K03164; -.
DR   OMA; SRWEVCL; -.
DR   OrthoDB; EOG73JKTM; -.
DR   PhylomeDB; P11388; -.
DR   TreeFam; TF105282; -.
DR   BRENDA; 5.99.1.3; 2681.
DR   Reactome; REACT_111214; G0 and Early G1.
DR   SignaLink; P11388; -.
DR   EvolutionaryTrace; P11388; -.
DR   GeneWiki; TOP2A; -.
DR   GenomeRNAi; 7153; -.
DR   NextBio; 27992; -.
DR   PRO; PR:P11388; -.
DR   Bgee; P11388; -.
DR   CleanEx; HS_TOP2A; -.
DR   ExpressionAtlas; P11388; baseline and differential.
DR   Genevestigator; P11388; -.
DR   GO; GO:0000793; C:condensed chromosome; IEA:Ensembl.
DR   GO; GO:0005737; C:cytoplasm; IEA:UniProtKB-KW.
DR   GO; GO:0009330; C:DNA topoisomerase complex (ATP-hydrolyzing); IDA:UniProtKB.
DR   GO; GO:0005730; C:nucleolus; IDA:UniProtKB.
DR   GO; GO:0005654; C:nucleoplasm; IDA:UniProtKB.
DR   GO; GO:0005634; C:nucleus; IDA:UniProtKB.
DR   GO; GO:0043234; C:protein complex; IDA:UniProtKB.
DR   GO; GO:0005524; F:ATP binding; IEA:UniProtKB-KW.
DR   GO; GO:0003682; F:chromatin binding; IDA:UniProtKB.
DR   GO; GO:0003677; F:DNA binding; IDA:UniProtKB.
DR   GO; GO:0008301; F:DNA binding, bending; IDA:UniProtKB.
DR   GO; GO:0003918; F:DNA topoisomerase type II (ATP-hydrolyzing) activity; IDA:UniProtKB.
DR   GO; GO:0008094; F:DNA-dependent ATPase activity; IDA:UniProtKB.
DR   GO; GO:0008144; F:drug binding; IDA:UniProtKB.
DR   GO; GO:0019899; F:enzyme binding; IPI:UniProtKB.
DR   GO; GO:0042826; F:histone deacetylase binding; IPI:UniProtKB.
DR   GO; GO:0000287; F:magnesium ion binding; IDA:UniProtKB.
DR   GO; GO:0044822; F:poly(A) RNA binding; IDA:UniProtKB.
DR   GO; GO:0008022; F:protein C-terminus binding; IPI:UniProtKB.
DR   GO; GO:0046982; F:protein heterodimerization activity; IPI:UniProtKB.
DR   GO; GO:0042803; F:protein homodimerization activity; IPI:UniProtKB.
DR   GO; GO:0005080; F:protein kinase C binding; IPI:UniProtKB.
DR   GO; GO:0043130; F:ubiquitin binding; IMP:UniProtKB.
DR   GO; GO:0030263; P:apoptotic chromosome condensation; IDA:UniProtKB.
DR   GO; GO:0006200; P:ATP catabolic process; IDA:GOC.
DR   GO; GO:0006974; P:cellular response to DNA damage stimulus; IDA:UniProtKB.
DR   GO; GO:0007059; P:chromosome segregation; IMP:UniProtKB.
DR   GO; GO:0006266; P:DNA ligation; IDA:UniProtKB.
DR   GO; GO:0006265; P:DNA topological change; IDA:UniProtKB.
DR   GO; GO:0006268; P:DNA unwinding involved in DNA replication; IBA:RefGenome.
DR   GO; GO:0040016; P:embryonic cleavage; IEA:Ensembl.
DR   GO; GO:0002244; P:hematopoietic progenitor cell differentiation; IEA:Ensembl.
DR   GO; GO:0000278; P:mitotic cell cycle; TAS:Reactome.
DR   GO; GO:0044774; P:mitotic DNA integrity checkpoint; IBA:RefGenome.
DR   GO; GO:0006312; P:mitotic recombination; IBA:RefGenome.
DR   GO; GO:0043065; P:positive regulation of apoptotic process; IDA:UniProtKB.
DR   GO; GO:0045870; P:positive regulation of single stranded viral RNA replication via double stranded DNA intermediate; IMP:UniProtKB.
DR   GO; GO:0045944; P:positive regulation of transcription from RNA polymerase II promoter; IEA:Ensembl.
DR   GO; GO:0045070; P:positive regulation of viral genome replication; IMP:UniProtKB.
DR   GO; GO:0000712; P:resolution of meiotic recombination intermediates; IBA:RefGenome.
DR   GO; GO:0000819; P:sister chromatid segregation; IBA:RefGenome.
DR   Gene3D; 1.10.268.10; -; 1.
DR   Gene3D; 3.30.1360.40; -; 1.
DR   Gene3D; 3.30.230.10; -; 1.
DR   Gene3D; 3.30.565.10; -; 1.
DR   Gene3D; 3.40.50.670; -; 1.
DR   Gene3D; 3.90.199.10; -; 1.
DR   InterPro; IPR024946; Arg_repress_C-like.
DR   InterPro; IPR012542; DTHCT.
DR   InterPro; IPR003594; HATPase_C.
DR   InterPro; IPR020568; Ribosomal_S5_D2-typ_fold.
DR   InterPro; IPR014721; Ribosomal_S5_D2-typ_fold_subgr.
DR   InterPro; IPR028466; Top2a.
DR   InterPro; IPR001241; Topo_IIA.
DR   InterPro; IPR002205; Topo_IIA_A/C.
DR   InterPro; IPR013758; Topo_IIA_A/C_ab.
DR   InterPro; IPR013757; Topo_IIA_A_a.
DR   InterPro; IPR013506; Topo_IIA_bsu_dom2.
DR   InterPro; IPR013759; Topo_IIA_cen_dom.
DR   InterPro; IPR013760; Topo_IIA_like_dom.
DR   InterPro; IPR018522; TopoIIA_CS.
DR   InterPro; IPR006171; Toprim_domain.
DR   PANTHER; PTHR10169:SF33; PTHR10169:SF33; 1.
DR   Pfam; PF00204; DNA_gyraseB; 1.
DR   Pfam; PF00521; DNA_topoisoIV; 1.
DR   Pfam; PF08070; DTHCT; 1.
DR   Pfam; PF02518; HATPase_c; 1.
DR   Pfam; PF01751; Toprim; 1.
DR   PRINTS; PR00418; TPI2FAMILY.
DR   SMART; SM00387; HATPase_c; 1.
DR   SMART; SM00433; TOP2c; 1.
DR   SMART; SM00434; TOP4c; 1.
DR   SUPFAM; SSF54211; SSF54211; 1.
DR   SUPFAM; SSF55874; SSF55874; 1.
DR   SUPFAM; SSF56719; SSF56719; 1.
DR   PROSITE; PS00177; TOPOISOMERASE_II; 1.
DR   PROSITE; PS50880; TOPRIM; 1.
PE   1: Evidence at protein level;
KW   3D-structure; Acetylation; Alternative splicing; ATP-binding;
KW   Complete proteome; Cytoplasm; Direct protein sequencing; DNA-binding;
KW   Isomerase; Magnesium; Metal-binding; Nucleotide-binding; Nucleus;
KW   Phosphoprotein; Polymorphism; Reference proteome; Topoisomerase.
FT   CHAIN         1   1531       DNA topoisomerase 2-alpha.
FT                                /FTId=PRO_0000145363.
FT   DOMAIN      455    572       Toprim. {ECO:0000255|PROSITE-
FT                                ProRule:PRU00995}.
FT   NP_BIND     148    150       ATP.
FT   NP_BIND     161    168       ATP.
FT   NP_BIND     376    378       ATP.
FT   REGION      342    344       Interaction with DNA. {ECO:0000305}.
FT   REGION      990    999       Interaction with DNA.
FT   MOTIF      1018   1028       Nuclear export signal.
FT   ACT_SITE    805    805       O-(5'-phospho-DNA)-tyrosine intermediate.
FT                                {ECO:0000250}.
FT   METAL       461    461       Magnesium 1; catalytic.
FT                                {ECO:0000255|PROSITE-ProRule:PRU00995}.
FT   METAL       541    541       Magnesium 1; catalytic.
FT                                {ECO:0000255|PROSITE-ProRule:PRU00995}.
FT   METAL       541    541       Magnesium 2.
FT   METAL       543    543       Magnesium 2.
FT   BINDING      91     91       ATP.
FT   BINDING     120    120       ATP.
FT   SITE        489    489       Interaction with DNA.
FT                                {ECO:0000255|PROSITE-ProRule:PRU00995}.
FT   SITE        492    492       Interaction with DNA.
FT   SITE        661    661       Interaction with DNA.
FT   SITE        662    662       Interaction with DNA.
FT   SITE        723    723       Interaction with DNA.
FT   SITE        757    757       Interaction with DNA.
FT   SITE        763    763       Interaction with DNA.
FT   SITE        804    804       Transition state stabilizer.
FT                                {ECO:0000250}.
FT   SITE        856    856       Important for DNA bending; intercalates
FT                                between base pairs of target DNA.
FT                                {ECO:0000250}.
FT   SITE        931    931       Interaction with DNA.
FT   MOD_RES       1      1       N-acetylmethionine.
FT                                {ECO:0000269|PubMed:20068231,
FT                                ECO:0000269|PubMed:21406692,
FT                                ECO:0000269|PubMed:22814378,
FT                                ECO:0000269|Ref.10}.
FT   MOD_RES       4      4       Phosphoserine.
FT                                {ECO:0000269|PubMed:17081983,
FT                                ECO:0000269|PubMed:18691976,
FT                                ECO:0000269|PubMed:20068231,
FT                                ECO:0000269|PubMed:21406692}.
FT   MOD_RES     282    282       Phosphothreonine.
FT                                {ECO:0000269|PubMed:20068231}.
FT   MOD_RES    1106   1106       Phosphoserine; by CK1.
FT                                {ECO:0000269|PubMed:17081983,
FT                                ECO:0000269|PubMed:18669648,
FT                                ECO:0000269|PubMed:19043076,
FT                                ECO:0000269|PubMed:20068231,
FT                                ECO:0000269|PubMed:21406692,
FT                                ECO:0000269|Ref.11}.
FT   MOD_RES    1205   1205       Phosphothreonine.
FT                                {ECO:0000269|PubMed:20068231}.
FT   MOD_RES    1213   1213       Phosphoserine.
FT                                {ECO:0000269|PubMed:16964243,
FT                                ECO:0000269|PubMed:17081983,
FT                                ECO:0000269|PubMed:18669648,
FT                                ECO:0000269|PubMed:18691976,
FT                                ECO:0000269|PubMed:20068231,
FT                                ECO:0000269|PubMed:21406692}.
FT   MOD_RES    1247   1247       Phosphoserine.
FT                                {ECO:0000269|PubMed:17081983,
FT                                ECO:0000269|PubMed:17924679,
FT                                ECO:0000269|PubMed:18691976,
FT                                ECO:0000269|PubMed:19690332,
FT                                ECO:0000269|PubMed:20068231,
FT                                ECO:0000269|PubMed:21406692}.
FT   MOD_RES    1295   1295       Phosphoserine.
FT                                {ECO:0000269|PubMed:21406692}.
FT   MOD_RES    1297   1297       Phosphoserine.
FT                                {ECO:0000269|PubMed:21406692}.
FT   MOD_RES    1299   1299       Phosphoserine.
FT                                {ECO:0000269|PubMed:21406692}.
FT   MOD_RES    1302   1302       Phosphoserine.
FT                                {ECO:0000269|PubMed:21406692}.
FT   MOD_RES    1332   1332       Phosphoserine.
FT                                {ECO:0000269|PubMed:17081983,
FT                                ECO:0000269|PubMed:18669648,
FT                                ECO:0000269|PubMed:18691976,
FT                                ECO:0000269|PubMed:20068231,
FT                                ECO:0000269|PubMed:21406692}.
FT   MOD_RES    1337   1337       Phosphoserine.
FT                                {ECO:0000269|PubMed:17081983,
FT                                ECO:0000269|PubMed:18669648,
FT                                ECO:0000269|PubMed:18691976,
FT                                ECO:0000269|PubMed:20068231,
FT                                ECO:0000269|PubMed:21406692}.
FT   MOD_RES    1343   1343       Phosphothreonine; by PLK3.
FT                                {ECO:0000269|PubMed:17081983,
FT                                ECO:0000269|PubMed:18062778,
FT                                ECO:0000269|PubMed:18669648,
FT                                ECO:0000269|PubMed:20068231,
FT                                ECO:0000269|PubMed:21406692}.
FT   MOD_RES    1351   1351       Phosphoserine.
FT                                {ECO:0000269|PubMed:17081983,
FT                                ECO:0000269|PubMed:18669648,
FT                                ECO:0000269|PubMed:20068231,
FT                                ECO:0000269|PubMed:21406692}.
FT   MOD_RES    1354   1354       Phosphoserine.
FT                                {ECO:0000269|PubMed:17081983,
FT                                ECO:0000269|PubMed:20068231,
FT                                ECO:0000269|PubMed:21406692}.
FT   MOD_RES    1374   1374       Phosphoserine.
FT                                {ECO:0000269|PubMed:16964243,
FT                                ECO:0000269|PubMed:17081983,
FT                                ECO:0000269|PubMed:19690332,
FT                                ECO:0000269|PubMed:20068231,
FT                                ECO:0000269|PubMed:21406692}.
FT   MOD_RES    1377   1377       Phosphoserine.
FT                                {ECO:0000269|PubMed:16964243,
FT                                ECO:0000269|PubMed:17081983,
FT                                ECO:0000269|PubMed:18691976,
FT                                ECO:0000269|PubMed:20068231,
FT                                ECO:0000269|PubMed:21406692}.
FT   MOD_RES    1387   1387       Phosphoserine.
FT                                {ECO:0000269|PubMed:19690332}.
FT   MOD_RES    1392   1392       Phosphoserine.
FT                                {ECO:0000269|PubMed:18669648}.
FT   MOD_RES    1393   1393       Phosphoserine.
FT                                {ECO:0000269|PubMed:18669648,
FT                                ECO:0000269|PubMed:19690332}.
FT   MOD_RES    1422   1422       N6-acetyllysine. {ECO:0000250}.
FT   MOD_RES    1442   1442       N6-acetyllysine. {ECO:0000250}.
FT   MOD_RES    1449   1449       Phosphoserine.
FT                                {ECO:0000269|PubMed:18669648}.
FT   MOD_RES    1469   1469       Phosphoserine; by CK2.
FT                                {ECO:0000269|PubMed:10942766,
FT                                ECO:0000269|PubMed:18669648,
FT                                ECO:0000269|PubMed:21406692}.
FT   MOD_RES    1470   1470       Phosphothreonine.
FT                                {ECO:0000269|PubMed:18669648,
FT                                ECO:0000269|PubMed:21406692}.
FT   MOD_RES    1471   1471       Phosphoserine.
FT                                {ECO:0000269|PubMed:18669648,
FT                                ECO:0000269|PubMed:20068231,
FT                                ECO:0000269|PubMed:21406692}.
FT   MOD_RES    1474   1474       Phosphoserine.
FT                                {ECO:0000269|PubMed:18669648,
FT                                ECO:0000269|PubMed:20068231,
FT                                ECO:0000269|PubMed:21406692}.
FT   MOD_RES    1476   1476       Phosphoserine.
FT                                {ECO:0000269|PubMed:21406692}.
FT   MOD_RES    1495   1495       Phosphoserine.
FT                                {ECO:0000269|PubMed:20068231}.
FT   MOD_RES    1504   1504       Phosphoserine.
FT                                {ECO:0000269|PubMed:18220336,
FT                                ECO:0000269|PubMed:19690332,
FT                                ECO:0000269|PubMed:20068231}.
FT   MOD_RES    1525   1525       Phosphoserine.
FT                                {ECO:0000269|PubMed:16964243,
FT                                ECO:0000269|PubMed:17081983,
FT                                ECO:0000269|PubMed:18669648,
FT                                ECO:0000269|PubMed:19690332,
FT                                ECO:0000269|PubMed:20068231,
FT                                ECO:0000269|PubMed:21406692}.
FT   VAR_SEQ     321    321       K -> KSSKYWSSRKSKQHILLNFFVLFKFINDAFFGICPF
FT                                K (in isoform 3). {ECO:0000303|Ref.8}.
FT                                /FTId=VSP_006529.
FT   VAR_SEQ     355    355       Q -> QRELCNGAILAHCNLRLMGSSDSPASASRVAGIAGG
FT                                CHHTQLIFVFLVETGFHHVGQAGLERLTSGDPPASASQSSG
FT                                ITDVK (in isoform 4).
FT                                {ECO:0000303|Ref.8}.
FT                                /FTId=VSP_006530.
FT   VAR_SEQ     401    401       A -> AHLYSRFLIDPFFPNMIPNMIFSFSKA (in
FT                                isoform 2). {ECO:0000303|Ref.8}.
FT                                /FTId=VSP_006531.
FT   VARIANT     450    450       R -> Q (in teniposide (VM-26) resistant
FT                                cells). {ECO:0000269|PubMed:1652758}.
FT                                /FTId=VAR_007532.
FT   VARIANT     487    487       R -> K (in amsacrine resistant cells).
FT                                {ECO:0000269|PubMed:1651812}.
FT                                /FTId=VAR_007533.
FT   VARIANT    1324   1324       T -> K (in dbSNP:rs28969502).
FT                                /FTId=VAR_029245.
FT   VARIANT    1386   1386       G -> D (in dbSNP:rs34300454).
FT                                /FTId=VAR_052594.
FT   VARIANT    1515   1515       A -> S (in dbSNP:rs11540720).
FT                                /FTId=VAR_052595.
FT   MUTAGEN     342    344       KKK->AAA: Reduced enzyme activity;
FT                                abolishes stimulation of ATPase activity
FT                                upon DNA binding.
FT                                {ECO:0000269|PubMed:23022727}.
FT   MUTAGEN     342    344       KKK->EEE: Strongly reduced enzyme
FT                                activity; abolishes stimulation of ATPase
FT                                activity upon DNA binding.
FT                                {ECO:0000269|PubMed:23022727}.
FT   MUTAGEN     461    461       E->A,C: Impairs bending of target DNA.
FT                                Strongly reduced DNA cleavage.
FT                                {ECO:0000269|PubMed:19697956,
FT                                ECO:0000269|PubMed:22323612}.
FT   MUTAGEN     541    541       D->A,C: Impairs bending of target DNA.
FT                                Strongly reduced DNA cleavage.
FT                                {ECO:0000269|PubMed:19697956,
FT                                ECO:0000269|PubMed:22323612}.
FT   MUTAGEN     543    543       D->A,C: Impairs bending of target DNA.
FT                                Strongly reduced DNA cleavage.
FT                                {ECO:0000269|PubMed:19697956,
FT                                ECO:0000269|PubMed:22323612}.
FT   MUTAGEN     545    545       D->A,C: Strongly reduced DNA cleavage.
FT                                {ECO:0000269|PubMed:19697956}.
FT   MUTAGEN    1469   1469       S->A: Abolishes binding to the antibody
FT                                MPM2. {ECO:0000269|PubMed:10942766}.
FT   CONFLICT    152    152       D -> H (in Ref. 4; CAA09762).
FT                                {ECO:0000305}.
FT   CONFLICT    180    180       E -> Q (in Ref. 4; CAA09762).
FT                                {ECO:0000305}.
FT   CONFLICT    327    327       D -> H (in Ref. 4; CAA09762).
FT                                {ECO:0000305}.
FT   CONFLICT   1022   1022       F -> L (in Ref. 4; CAA09762).
FT                                {ECO:0000305}.
FT   CONFLICT   1274   1274       T -> S (in Ref. 4; CAA09762).
FT                                {ECO:0000305}.
FT   CONFLICT   1295   1295       S -> P (in Ref. 1; AAA61209).
FT                                {ECO:0000305}.
FT   HELIX        30     33
FT   STRAND       34     36
FT   HELIX        39     45
FT   HELIX        48     51
FT   STRAND       57     65
FT   TURN         66     68
FT   STRAND       69     77
FT   HELIX        79     98
FT   STRAND      104    110
FT   TURN        111    114
FT   STRAND      115    123
FT   STRAND      128    130
FT   TURN        131    134
FT   HELIX       137    143
FT   STRAND      144    149
FT   HELIX       153    155
FT   HELIX       166    172
FT   STRAND      174    183
FT   TURN        184    187
FT   STRAND      188    195
FT   TURN        196    199
FT   STRAND      205    208
FT   STRAND      214    221
FT   HELIX       223    226
FT   HELIX       233    249
FT   STRAND      250    252
FT   STRAND      254    257
FT   HELIX       267    275
FT   STRAND      281    285
FT   STRAND      289    294
FT   STRAND      297    303
FT   STRAND      305    307
FT   STRAND      309    314
FT   HELIX       324    341
FT   HELIX       353    357
FT   STRAND      360    366
FT   STRAND      373    375
FT   HELIX       385    387
FT   STRAND      388    390
FT   HELIX       396    402
FT   HELIX       411    415
FT   STRAND      419    421
FT   TURN        445    448
FT   HELIX       452    454
FT   STRAND      456    462
FT   HELIX       463    476
FT   STRAND      478    486
FT   HELIX       498    503
FT   HELIX       505    514
FT   STRAND      518    520
FT   HELIX       525    530
FT   STRAND      534    539
FT   HELIX       544    560
FT   HELIX       562    566
FT   STRAND      570    573
FT   STRAND      577    581
FT   STRAND      586    590
FT   HELIX       592    601
FT   STRAND      607    612
FT   HELIX       616    618
FT   HELIX       621    629
FT   HELIX       631    634
FT   STRAND      635    639
FT   HELIX       644    653
FT   HELIX       658    678
FT   STRAND      685    688
FT   STRAND      691    694
FT   HELIX       695    701
FT   HELIX       703    714
FT   TURN        718    720
FT   HELIX       724    735
FT   HELIX       744    754
FT   HELIX       762    772
FT   TURN        793    799
FT   TURN        803    805
FT   HELIX       812    817
FT   HELIX       822    824
FT   HELIX       831    833
FT   TURN        848    850
FT   STRAND      853    856
FT   STRAND      861    864
FT   HELIX       869    881
FT   STRAND      897    903
FT   STRAND      906    910
FT   STRAND      921    923
FT   HELIX       932    938
FT   HELIX       940    944
FT   STRAND      948    950
FT   STRAND      955    959
FT   STRAND      968    971
FT   HELIX       974    983
FT   HELIX       985    988
FT   STRAND      992    996
FT   STRAND     1000   1003
FT   STRAND     1009   1011
FT   HELIX      1015   1059
FT   HELIX      1070   1080
FT   HELIX      1086   1091
FT   HELIX      1126   1129
FT   HELIX      1138   1160
FT   HELIX      1163   1189
SQ   SEQUENCE   1531 AA;  174385 MW;  3DF40BC9E84789DC CRC64;
     MEVSPLQPVN ENMQVNKIKK NEDAKKRLSV ERIYQKKTQL EHILLRPDTY IGSVELVTQQ
     MWVYDEDVGI NYREVTFVPG LYKIFDEILV NAADNKQRDP KMSCIRVTID PENNLISIWN
     NGKGIPVVEH KVEKMYVPAL IFGQLLTSSN YDDDEKKVTG GRNGYGAKLC NIFSTKFTVE
     TASREYKKMF KQTWMDNMGR AGEMELKPFN GEDYTCITFQ PDLSKFKMQS LDKDIVALMV
     RRAYDIAGST KDVKVFLNGN KLPVKGFRSY VDMYLKDKLD ETGNSLKVIH EQVNHRWEVC
     LTMSEKGFQQ ISFVNSIATS KGGRHVDYVA DQIVTKLVDV VKKKNKGGVA VKAHQVKNHM
     WIFVNALIEN PTFDSQTKEN MTLQPKSFGS TCQLSEKFIK AAIGCGIVES ILNWVKFKAQ
     VQLNKKCSAV KHNRIKGIPK LDDANDAGGR NSTECTLILT EGDSAKTLAV SGLGVVGRDK
     YGVFPLRGKI LNVREASHKQ IMENAEINNI IKIVGLQYKK NYEDEDSLKT LRYGKIMIMT
     DQDQDGSHIK GLLINFIHHN WPSLLRHRFL EEFITPIVKV SKNKQEMAFY SLPEFEEWKS
     STPNHKKWKV KYYKGLGTST SKEAKEYFAD MKRHRIQFKY SGPEDDAAIS LAFSKKQIDD
     RKEWLTNFME DRRQRKLLGL PEDYLYGQTT TYLTYNDFIN KELILFSNSD NERSIPSMVD
     GLKPGQRKVL FTCFKRNDKR EVKVAQLAGS VAEMSSYHHG EMSLMMTIIN LAQNFVGSNN
     LNLLQPIGQF GTRLHGGKDS ASPRYIFTML SSLARLLFPP KDDHTLKFLY DDNQRVEPEW
     YIPIIPMVLI NGAEGIGTGW SCKIPNFDVR EIVNNIRRLM DGEEPLPMLP SYKNFKGTIE
     ELAPNQYVIS GEVAILNSTT IEISELPVRT WTQTYKEQVL EPMLNGTEKT PPLITDYREY
     HTDTTVKFVV KMTEEKLAEA ERVGLHKVFK LQTSLTCNSM VLFDHVGCLK KYDTVLDILR
     DFFELRLKYY GLRKEWLLGM LGAESAKLNN QARFILEKID GKIIIENKPK KELIKVLIQR
     GYDSDPVKAW KEAQQKVPDE EENEESDNEK ETEKSDSVTD SGPTFNYLLD MPLWYLTKEK
     KDELCRLRNE KEQELDTLKR KSPSDLWKED LATFIEELEA VEAKEKQDEQ VGLPGKGGKA
     KGKKTQMAEV LPSPRGQRVI PRITIEMKAE AEKKNKKKIK NENTEGSPQE DGVELEGLKQ
     RLEKKQKREP GTKTKKQTTL AFKPIKKGKK RNPWSDSESD RSSDESNFDV PPRETEPRRA
     ATKTKFTMDL DSDEDFSDFD EKTDDEDFVP SDASPPKTKT SPKLSNKELK PQKSVVSDLE
     ADDVKGSVPL SSSPPATHFP DETEITNPVP KKNVTVKKTA AKSQSSTSTT GAKKRAAPKG
     TKRDPALNSG VSQKPDPAKT KNRRKRKPST SDDSDSNFEK IVSKAVTSKK SKGESDDFHM
     DFDSAVAPRA KSVRAKKPIK YLEESDEDDL F
```

**Mascot:** http://www.matrixscience.com/
