# Supplementary material for: Non-SMC Element 2 (NSMCE2) of the SMC5/6 Complex Helps to Resolve Topological Stress
Source: Int J Mol Sci. 2016 Oct 26;17(11):1782. doi: 10.3390/ijms17111782 (PMC5133783; doi:10.3390/ijms17111782)
Supplement: Supplementary file 1 [file ijms-17-01782-s001.zip › ijms-142388-Supplementary Materials/ijms-142388 Data S1-S3.zip!/ijms-142388 Data S1-S3/Supplementary Data 2.htm]

../data/20141126/FTgmiaTTO.dat (Mascot Search Results)


# MASCOT Search Results

Search metadata

|  |  |  |
| --- | --- | --- |
| User | : | Dave |
| E-mail | : | d.speijer@amc.uva.nl |
| MS data file | : | ext1861\_E7\_300\_RT.mgf |
| Database | : | SwissProt 2014\_10 (546,790 sequences; 194,613,039 residues) |
| Timestamp | : | 26 Nov 2014 at 14:44:45 GMT |

Repeat search

All

Non-significant

Unassigned

[help]

Export


As
XML
CSV
pepXML
mzIdentML
mzTab
DTASelect
Mascot DAT File
MGF Peak List

Not what you expected? Try the peptide summary.

Search parameters

|  |  |  |
| --- | --- | --- |
| Type of search | : | MS/MS Ion Search |
| Enzyme | : | Trypsin |
| Fixed modifications | : | Carbamidomethyl (C) |
| Variable modifications | : | Oxidation (M) |
| Mass values | : | Monoisotopic |
| Protein mass | : | Unrestricted |
| Peptide mass tolerance | : | ± 0.3 Da |
| Fragment mass tolerance | : | ± 0.4 Da |
| Max missed cleavages | : | 1 |
| Instrument type | : | ESI-TRAP |
| Number of queries | : | 300 |

### Score distribution

**Peptide score distribution.** Ions score is −10log(*P*), where *P* is the probability that the observed match is a random event.

There are **40** peptide matches above identity threshold and **50** matches above homology threshold for **300** queries. Histogram score range is (0, 105). On average, individual ions scores **> 41** (beyond green shading) indicate **identity or extensive homology** (p<0.05).

**[Deprecated]** Protein score distribution.
Score distribution for family members in the first 50 proteins.
Protein scores are derived from ions scores as a non-probabilistic basis for
ranking protein families.

Modification statistics

| Modification | Site | Above thr. |
| --- | --- | --- |
| Oxidation | M | 12 |
| Carbamidomethyl | C | 2 |

Legend

| Dupes | Expect | Rank | U | 1 | 2 | Peptide |  |
| --- | --- | --- | --- | --- | --- | --- | --- |
|  | 0.037 | 2 |  |  |  | GAYSLSLR | significant |
|  | 9 | 1 |  |  |  | GFFLFVEGGR | top ranking |
|  | 6.4e-05 | 1 |  |  |  | GSSIFGLAPGK | significant and top ranking |
|  | 1.3e-06 | 1 |  |  |  | SSGTSYPDVLK | peptide is found in all proteins in family member 1 |
|  | 6.2e-07 | 1 |  |  |  | VCNYVSWIK | peptide is found in some but not all proteins in family member 2 |
|  | 6.4e-05 | 1 | U |  |  | GSSIFGLAPGK | unique |
| 2 | 5.7e-05 | 1 |  |  |  | LNTLETEEWFFK | peptide has two duplicates |
|  | 0.18 | 1 |  |  |  | LNTLETEEWFFK | duplicate peptide |

Right-facing triangle () in the Dupes or Rank column indicates content that can be expanded by clicking on it. Down-facing triangle () indicates the content is expanded and can be collapsed. For more details about particular columns, see results format help.

## Protein Family Summary

Filters

Significance threshold p<


Ions score or expect cut-off

Max. number of families


Dendrograms cut at

[help]

Taxonomy

Preferred taxonomy
:   All entries
    . . Archaea (Archaeobacteria)
    . . Eukaryota (eucaryotes)
    . . . . Alveolata (alveolates)
    . . . . . . Plasmodium falciparum (malaria parasite)
    . . . . . . Other Alveolata
    . . . . Metazoa (Animals)
    . . . . . . Caenorhabditis elegans
    . . . . . . Drosophila (fruit flies)
    . . . . . . Chordata (vertebrates and relatives)
    . . . . . . . . bony vertebrates
    . . . . . . . . . . lobe-finned fish and tetrapod clade
    . . . . . . . . . . . . Mammalia (mammals)
    . . . . . . . . . . . . . . Primates
    . . . . . . . . . . . . . . . . Homo sapiens (human)
    . . . . . . . . . . . . . . . . Other primates
    . . . . . . . . . . . . . . Rodentia (Rodents)
    . . . . . . . . . . . . . . . . Mus.
    . . . . . . . . . . . . . . . . . . Mus musculus (house mouse)
    . . . . . . . . . . . . . . . . Rattus
    . . . . . . . . . . . . . . . . Other rodentia
    . . . . . . . . . . . . . . Other mammalia
    . . . . . . . . . . . . Xenopus laevis (African clawed frog)
    . . . . . . . . . . . . Other lobe-finned fish and tetrapod clade
    . . . . . . . . . . Actinopterygii (ray-finned fishes)
    . . . . . . . . . . . . Takifugu rubripes (Japanese Pufferfish)
    . . . . . . . . . . . . Danio rerio (zebra fish)
    . . . . . . . . . . . . Other Actinopterygii
    . . . . . . . . Other Chordata
    . . . . . . Other Metazoa
    . . . . Dictyostelium discoideum
    . . . . Fungi
    . . . . . . Saccharomyces Cerevisiae (baker's yeast)
    . . . . . . Schizosaccharomyces pombe (fission yeast)
    . . . . . . Pneumocystis carinii
    . . . . . . Other Fungi
    . . . . Viridiplantae (Green Plants)
    . . . . . . Arabidopsis thaliana (thale cress)
    . . . . . . Oryza sativa (rice)
    . . . . . . Other green plants
    . . . . Other Eukaryota
    . . Bacteria (Eubacteria)
    . . . . Actinobacteria (class)
    . . . . . . Mycobacterium tuberculosis complex
    . . . . . . Other Actinobacteria (class)
    . . . . Firmicutes (gram-positive bacteria)
    . . . . . . Bacillus subtilis
    . . . . . . Mycoplasma
    . . . . . . Streptococcus Pneumoniae
    . . . . . . Streptomyces coelicolor
    . . . . . . Other Firmicutes
    . . . . Proteobacteria (purple bacteria)
    . . . . . . Agrobacterium tumefaciens
    . . . . . . Campylobacter jejuni
    . . . . . . Escherichia coli
    . . . . . . Neisseria meningitidis
    . . . . . . Salmonella
    . . . . . . Other Proteobacteria
    . . . . Other Bacteria
    . . Viruses
    . . . . Hepatitis C virus
    . . . . Other viruses
    . . Other (includes plasmids and artificial sequences)
    . . unclassified
    . . Species information unavailable

Show

- Proteins (8)
- Report Builder
- Unassigned (223)

## Protein families 1–8 (out of 8)

---

1
5
10
20
50
100
200
300
400
500
600
700
800
900
1000
1500
2000
all
per page

Page: 1

|  |  |  |
| --- | --- | --- |
|  | Accession Description contains starts with is equal to | |
|  | Family Page | |
|  | Query | |
|  | Observed Mr(expt) Mr(calc) | |
|  | Sequence contains starts with is equal to | |
|  | Fixed mod <select> Carbamidomethyl (C) | |
|  | Variable mod <select> Oxidation (M) | |

### 1

|  |  | Accession | Score | Description |
| --- | --- | --- | --- | --- |
|  | 1 | TOP2A\_HUMAN | 542 | DNA topoisomerase 2-alpha OS=Homo sapiens GN=TOP2A PE=1 SV=3 |

### 2

|  |  | Accession | Score | Description |
| --- | --- | --- | --- | --- |
|  | 1 | K1C10\_HUMAN | 209 | Keratin, type I cytoskeletal 10 OS=Homo sapiens GN=KRT10 PE=1 SV=6 |

### 3

|  |  | Accession | Score | Description |
| --- | --- | --- | --- | --- |
|  | 1 | K2C1\_HUMAN | 204 | Keratin, type II cytoskeletal 1 OS=Homo sapiens GN=KRT1 PE=1 SV=6 |

### 4

|  |  | Accession | Score | Description |
| --- | --- | --- | --- | --- |
|  | 1 | TRY1\_BOVIN | 197 | Cationic trypsin OS=Bos taurus PE=1 SV=3 |

### 5

|  |  | Accession | Score | Description |
| --- | --- | --- | --- | --- |
|  | 1 | K1C9\_HUMAN | 86 | Keratin, type I cytoskeletal 9 OS=Homo sapiens GN=KRT9 PE=1 SV=3 |

### 6

|  |  | Accession | Score | Description |
| --- | --- | --- | --- | --- |
|  | 1 | THIO\_ECOLI | 71 | Thioredoxin-1 OS=Escherichia coli (strain K12) GN=trxA PE=1 SV=2 |

### 7

|  |  | Accession | Score | Description |
| --- | --- | --- | --- | --- |
|  | 1 | IGHG\_RABIT | 37 | Ig gamma chain C region OS=Oryctolagus cuniculus PE=1 SV=1 |

### 8

|  |  | Accession | Score | Description |
| --- | --- | --- | --- | --- |
|  | 1 | EFTS\_BREBN | 21 | Elongation factor Ts OS=Brevibacillus brevis (strain 47 / JCM 6285 / NBRC 100599) GN=tsf PE=3 SV=1 |

1
5
10
20
50
100
200
300
400
500
600
700
800
900
1000
1500
2000
all
per page

Page: 1

---

Not what you expected? Try the peptide summary.

**Mascot:** http://www.matrixscience.com/

<xsl:stylesheet version="1.0" xmlns:xsl="http://www.w3.org/1999/XSL/Transform" xmlns:cc="http://www.matrixscience.com/xmlns/schema/cc" xmlns:tc="http://www.matrixscience.com/xmlns/schema/tc" xmlns:html="http://www.w3.org/1999/xhtml" > <xsl:output method="xml" version="1.0" indent="no" standalone="yes" omit-xml-declaration="yes" cdata-section-elements="script pre style" /> <xsl:param name="xml-base-supported">1</xsl:param> <xsl:template match="@\*|node()"> <xsl:copy> <xsl:apply-templates select="@\*|node()"/> </xsl:copy> </xsl:template> <xsl:param name="state" select="'default'"/> <xsl:template match="/tc:data"> <xsl:copy> <xsl:apply-templates select="@\*|node()"/> </xsl:copy> </xsl:template> <xsl:template match="/tc:data/tc:elements"> <xsl:if test="@state=$state"> <xsl:copy> <xsl:apply-templates select="@\*|node()"/> </xsl:copy> </xsl:if> </xsl:template> <xsl:template match="tc:\*"> <xsl:copy> <xsl:apply-templates select="@\*|node()"/> </xsl:copy> </xsl:template> <xsl:template match="tc:elements/html:\*"> <xsl:copy> <xsl:copy-of select="./ancestor-or-self::\*/@xml:base"/> <xsl:call-template name="massage-html-element"/> </xsl:copy> </xsl:template> <xsl:template match="html:\*"> <xsl:copy> <xsl:call-template name="massage-html-element"/> </xsl:copy> </xsl:template> <xsl:template name="massage-html-element"> <xsl:choose> <xsl:when test="@href or @src or @action or @data"> <xsl:copy-of select="./ancestor-or-self::\*/@xml:base"/> <xsl:call-template name="massage-uri"> <xsl:with-param name="base"><xsl:value-of select="./ancestor::\*/@xml:base"/></xsl:with-param> </xsl:call-template> <xsl:apply-templates select="@\*[local-name() != 'href' and local-name() != 'src' and local-name() != 'action' and local-name() != 'data']|node()"/> </xsl:when> <xsl:when test="local-name()='param' and @name='src'"> <xsl:copy-of select="./ancestor-or-self::\*/@xml:base"/> <xsl:apply-templates select="@\*|node()"/> </xsl:when> <xsl:otherwise> <xsl:apply-templates select="@\*|node()"/> </xsl:otherwise> </xsl:choose> <xsl:if test="not(contains(' area base basefont br col frame hr img input isindex links meta param ', concat(' ', normalize-space(local-name()), ' ')))"> <xsl:if test="count(./\*) = 0 and (string-length(./child::text()) = 0 or normalize-space(./child::text()) = ' ' or normalize-space(./child::text()) = '')"> <xsl:text>&#10;</xsl:text> </xsl:if> </xsl:if> </xsl:template> <xsl:template name="massage-uri"> <xsl:param name="base"/> <xsl:variable name="uri-attribute"> <xsl:choose> <xsl:when test="@href">href</xsl:when> <xsl:when test="@src">src</xsl:when> <xsl:when test="@action">action</xsl:when> <xsl:when test="@data">data</xsl:when> </xsl:choose> </xsl:variable> <xsl:variable name="uri-value"> <xsl:choose> <xsl:when test="@href"><xsl:value-of select="@href"/></xsl:when> <xsl:when test="@src"><xsl:value-of select="@src"/></xsl:when> <xsl:when test="@action"><xsl:value-of select="@action"/></xsl:when> <xsl:when test="@data"><xsl:value-of select="@data"/></xsl:when> </xsl:choose> </xsl:variable> <xsl:choose> <xsl:when test="$base and $xml-base-supported != 1 and starts-with($base, 'http://') and $uri-attribute and starts-with($uri-value, '../')"> <xsl:choose> <xsl:when test="substring($base, string-length($base), 1) = '/'"> <xsl:attribute name="{$uri-attribute}"> <xsl:value-of select="concat($base, $uri-value)"/> </xsl:attribute> </xsl:when> <xsl:otherwise> <xsl:attribute name="{$uri-attribute}"><xsl:value-of select="concat($base, '/', $uri-value)"/></xsl:attribute> </xsl:otherwise> </xsl:choose> </xsl:when> <xsl:otherwise> <xsl:attribute name="{$uri-attribute}"><xsl:value-of select="$uri-value"/></xsl:attribute> </xsl:otherwise> </xsl:choose> </xsl:template> <xsl:template match="@cc:in"/> <xsl:variable name="IE" select="system-property('xsl:vendor')='Microsoft'"/> <xsl:template match="\*[@cc:in='!IE']"> <xsl:if test="not($IE)"> <xsl:copy> <xsl:apply-templates select="@\*|node()"/> </xsl:copy> </xsl:if> </xsl:template> <xsl:template match="\*[@cc:in and not(contains(@cc:in, '!IE'))]"> <xsl:if test="$IE"> <xsl:comment><![CDATA[[if IE]><![if ]]><xsl:value-of select="@cc:in"/><![CDATA[]><![endif]]]></xsl:comment> <xsl:copy> <xsl:apply-templates select="@\*|node()"/> </xsl:copy> <xsl:comment><![CDATA[[if IE]><![endif]><![endif]]]></xsl:comment> </xsl:if> </xsl:template></xsl:stylesheet>
%3Cxsl%3Astylesheet%20version%3D%221.0%22%20%20%20%20xmlns%3Axsl%3D%22http%3A%2F%2Fwww.w3.org%2F1999%2FXSL%2FTransform%22%20%20%20%20xmlns%3Ahtml%3D%22http%3A%2F%2Fwww.w3.org%2F1999%2Fxhtml%22%20%20%20%20xmlns%3D%22http%3A%2F%2Fwww.w3.org%2F1999%2Fxhtml%22%20%20%20%20id%3D%22FloatingTableCells%22%20%20%20%20%3E%20%3Cxsl%3Apreserve-space%20elements%3D%22%2A%22%2F%3E%20%3Cxsl%3Atemplate%20match%3D%22%40%2A%7Cnode%28%29%22%3E%20%3Cxsl%3Acopy%3E%20%3Cxsl%3Aapply-templates%20select%3D%22%40%2A%7Cnode%28%29%22%2F%3E%20%3C%2Fxsl%3Acopy%3E%20%3C%2Fxsl%3Atemplate%3E%20%3Cxsl%3Atemplate%20match%3D%22%40%2A%7Cnode%28%29%22%20mode%3D%22screen-form%22%3E%20%3Cxsl%3Acopy%3E%20%3Cxsl%3Aapply-templates%20select%3D%22%40%2A%7Cnode%28%29%22%20mode%3D%22screen-form%22%2F%3E%20%3C%2Fxsl%3Acopy%3E%20%3C%2Fxsl%3Atemplate%3E%20%3Cxsl%3Atemplate%20match%3D%22%40%2A%7Cnode%28%29%22%20mode%3D%22print-form%22%3E%20%3Cxsl%3Acopy%3E%20%3Cxsl%3Aapply-templates%20select%3D%22%40%2A%7Cnode%28%29%22%20mode%3D%22print-form%22%2F%3E%20%3C%2Fxsl%3Acopy%3E%20%3C%2Fxsl%3Atemplate%3E%20%3Cxsl%3Atemplate%20match%3D%22%40%2A%7Cnode%28%29%22%20mode%3D%22screen-table%22%3E%20%3Cxsl%3Acopy%3E%20%3Cxsl%3Aapply-templates%20select%3D%22%40%2A%7Cnode%28%29%22%20mode%3D%22screen-table%22%2F%3E%20%3C%2Fxsl%3Acopy%3E%20%3C%2Fxsl%3Atemplate%3E%20%3Cxsl%3Akey%20%20%20%20%20%20%20%20%20name%3D%22is-floating-table%22%20%20%20%20%20%20%20%20%20match%3D%22html%3Atable%5Bcontains%28concat%28%27%20%27%2C%20normalize-space%28%40class%29%2C%20%27%20%27%29%2C%20%27%20has-floating-table-cells%20%27%29%5D%22%20%20%20%20%20%20%20%20%20use%3D%22generate-id%28%29%22%20%20%20%20%20%20%20%20%2F%3E%20%3Cxsl%3Atemplate%20match%3D%22html%3Aform%22%3E%20%3Cxsl%3Achoose%3E%20%3Cxsl%3Awhen%20test%3D%22.%2F%2Fhtml%3Atable%5Bkey%28%27is-floating-table%27%2C%20generate-id%28%29%29%5D%22%3E%20%3Cxsl%3Acopy%3E%20%3Cxsl%3Aapply-templates%20select%3D%22%40%2A%7Cnode%28%29%22%20mode%3D%22print-form%22%2F%3E%20%3C%2Fxsl%3Acopy%3E%20%3Cxsl%3Acopy%3E%20%3Cxsl%3Aapply-templates%20select%3D%22%40%2A%7Cnode%28%29%22%20mode%3D%22screen-form%22%2F%3E%20%3C%2Fxsl%3Acopy%3E%20%3C%2Fxsl%3Awhen%3E%20%3Cxsl%3Aotherwise%3E%20%3Cxsl%3Acopy%3E%20%3Cxsl%3Aapply-templates%20select%3D%22%40%2A%7Cnode%28%29%22%20%2F%3E%20%3C%2Fxsl%3Acopy%3E%20%3C%2Fxsl%3Aotherwise%3E%20%3C%2Fxsl%3Achoose%3E%20%3C%2Fxsl%3Atemplate%3E%20%3Cxsl%3Atemplate%20match%3D%22html%3Atable%5Bnot%28ancestor%3A%3Ahtml%3Aform%29%5D%22%3E%20%3Cxsl%3Achoose%3E%20%3Cxsl%3Awhen%20test%3D%22key%28%27is-floating-table%27%2C%20generate-id%28%29%29%22%3E%20%3Cxsl%3Acall-template%20name%3D%22create-print-table%22%2F%3E%20%3Cxsl%3Acall-template%20name%3D%22create-screen-table%22%2F%3E%20%3C%2Fxsl%3Awhen%3E%20%3Cxsl%3Aotherwise%3E%20%3Cxsl%3Acopy%3E%20%3Cxsl%3Aapply-templates%20select%3D%22%40%2A%7Cnode%28%29%22%2F%3E%20%3C%2Fxsl%3Acopy%3E%20%3C%2Fxsl%3Aotherwise%3E%20%3C%2Fxsl%3Achoose%3E%20%3C%2Fxsl%3Atemplate%3E%20%3Cxsl%3Atemplate%20match%3D%22html%3Atable%5Bancestor%3A%3Ahtml%3Aform%5D%22%20mode%3D%22screen-form%22%3E%20%3Cxsl%3Achoose%3E%20%3Cxsl%3Awhen%20test%3D%22key%28%27is-floating-table%27%2C%20generate-id%28%29%29%22%3E%20%3Cxsl%3Acall-template%20name%3D%22create-screen-table%22%2F%3E%20%3C%2Fxsl%3Awhen%3E%20%3Cxsl%3Aotherwise%3E%20%3Cxsl%3Acopy%3E%20%3Cxsl%3Aapply-templates%20select%3D%22%40%2A%7Cnode%28%29%22%20mode%3D%22screen-form%22%2F%3E%20%3C%2Fxsl%3Acopy%3E%20%3C%2Fxsl%3Aotherwise%3E%20%3C%2Fxsl%3Achoose%3E%20%3C%2Fxsl%3Atemplate%3E%20%3Cxsl%3Atemplate%20match%3D%22html%3Atable%5Bancestor%3A%3Ahtml%3Aform%5D%22%20mode%3D%22print-form%22%3E%20%3Cxsl%3Achoose%3E%20%3Cxsl%3Awhen%20test%3D%22key%28%27is-floating-table%27%2C%20generate-id%28%29%29%22%3E%20%3Cxsl%3Acall-template%20name%3D%22create-print-table%22%2F%3E%20%3C%2Fxsl%3Awhen%3E%20%3Cxsl%3Aotherwise%3E%20%3Cxsl%3Acopy%3E%20%3Cxsl%3Aapply-templates%20select%3D%22%40%2A%7Cnode%28%29%22%20mode%3D%22print-form%22%2F%3E%20%3C%2Fxsl%3Acopy%3E%20%3C%2Fxsl%3Aotherwise%3E%20%3C%2Fxsl%3Achoose%3E%20%3C%2Fxsl%3Atemplate%3E%20%3Cxsl%3Atemplate%20name%3D%22create-screen-table%22%3E%20%3Cxsl%3Acall-template%20name%3D%22create-table%22%3E%20%3Cxsl%3Awith-param%20name%3D%22table-type%22%20select%3D%22%27floating-table-cells-screen%27%22%2F%3E%20%3C%2Fxsl%3Acall-template%3E%20%3C%2Fxsl%3Atemplate%3E%20%3Cxsl%3Atemplate%20name%3D%22create-print-table%22%3E%20%3Cxsl%3Acall-template%20name%3D%22create-table%22%3E%20%3Cxsl%3Awith-param%20name%3D%22table-type%22%20select%3D%22%27floating-table-cells-print%27%22%2F%3E%20%3C%2Fxsl%3Acall-template%3E%20%3C%2Fxsl%3Atemplate%3E%20%3Cxsl%3Atemplate%20name%3D%22create-table%22%3E%20%3Cxsl%3Aparam%20name%3D%22table-type%22%2F%3E%20%3Cxsl%3Avariable%20name%3D%22full-class%22%20select%3D%22concat%28%27%20%27%2C%20normalize-space%28%40class%29%2C%20%27%20%27%29%22%2F%3E%20%3Cxsl%3Acopy%3E%20%3Cxsl%3Aapply-templates%20select%3D%22%40%2A%5Bname%28%29%20%21%3D%20%27class%27%5D%22%2F%3E%20%3Cxsl%3Aattribute%20name%3D%22class%22%3E%20%3Cxsl%3Achoose%3E%20%3Cxsl%3Awhen%20test%3D%22contains%28%24full-class%2C%20%27%20has-floating-table-cells%20%27%29%22%3E%20%3Cxsl%3Avalue-of%20select%3D%22normalize-space%28concat%28substring-before%28%24full-class%2C%20%27%20has-floating-table-cells%20%27%29%2C%20%27%20%27%2C%20substring-after%28%24full-class%2C%20%27%20has-floating-table-cells%20%27%29%2C%20%27%20%27%2C%20%24table-type%29%29%22%2F%3E%20%3C%2Fxsl%3Awhen%3E%20%3Cxsl%3Aotherwise%3E%20%3Cxsl%3Avalue-of%20select%3D%22normalize-space%28concat%28%24full-class%2C%20%27%20%27%2C%20%24table-type%29%29%22%2F%3E%20%3C%2Fxsl%3Aotherwise%3E%20%3C%2Fxsl%3Achoose%3E%20%3C%2Fxsl%3Aattribute%3E%20%3Cxsl%3Achoose%3E%20%3Cxsl%3Awhen%20test%3D%22%24table-type%20%3D%20%27floating-table-cells-screen%27%22%3E%20%3Cxsl%3Aapply-templates%20select%3D%22node%28%29%22%20mode%3D%22screen-table%22%2F%3E%20%3C%2Fxsl%3Awhen%3E%20%3Cxsl%3Aotherwise%3E%20%3Cxsl%3Aapply-templates%20select%3D%22node%28%29%22%2F%3E%20%3C%2Fxsl%3Aotherwise%3E%20%3C%2Fxsl%3Achoose%3E%20%3C%2Fxsl%3Acopy%3E%20%3C%2Fxsl%3Atemplate%3E%20%3Cxsl%3Akey%20%20%20%20%20%20%20%20%20name%3D%22floated-sibling%22%20%20%20%20%20%20%20%20%20match%3D%22html%3Atable%5Bcontains%28concat%28%27%20%27%2C%20normalize-space%28%40class%29%2C%20%27%20%27%29%2C%20%27%20has-floating-table-cells%20%27%29%5D%2Fhtml%3Atbody%2Fhtml%3Atr%5Bhtml%3Atd%5Bcontains%28concat%28%27%20%27%2C%20normalize-space%28%40class%29%2C%20%27%20%27%29%2C%20%27%20floating-table-cell%20%27%29%5D%5D%22%20%20%20%20%20%20%20%20%20use%3D%22generate-id%28preceding-sibling%3A%3Ahtml%3Atr%5B1%5D%29%22%20%20%20%20%20%20%20%20%2F%3E%20%3Cxsl%3Atemplate%20match%3D%22html%3Atr%22%20mode%3D%22screen-table%22%3E%20%3Cxsl%3Acopy%3E%20%3Cxsl%3Achoose%3E%20%3Cxsl%3Awhen%20test%3D%22count%28key%28%27floated-sibling%27%2C%20generate-id%28%29%29%29%22%3E%20%3Cxsl%3Aapply-templates%20select%3D%22%40%2A%22%20mode%3D%22screen-table%22%2F%3E%20%3Cxsl%3Avariable%20name%3D%22nodes%22%20select%3D%22key%28%27floated-sibling%27%2C%20generate-id%28%29%29%2Fhtml%3Atd%5Bcontains%28concat%28%27%20%27%2C%20normalize-space%28%40class%29%2C%20%27%20%27%29%2C%20%27%20floating-table-cell%20%27%29%5D%2Fnode%28%29%22%20%2F%3E%20%3Cxsl%3Afor-each%20select%3D%22%2A%22%3E%20%3Cxsl%3Acopy%3E%20%3Cxsl%3Achoose%3E%20%3Cxsl%3Awhen%20test%3D%22following-sibling%3A%3A%2A%22%3E%20%3Cxsl%3Aapply-templates%20select%3D%22%40%2A%7Cnode%28%29%22%20mode%3D%22screen-table%22%2F%3E%20%3C%2Fxsl%3Awhen%3E%20%3Cxsl%3Aotherwise%3E%20%3Cxsl%3Aapply-templates%20select%3D%22%40%2A%22%20mode%3D%22screen-table%22%2F%3E%20%3Cxsl%3Acopy-of%20select%3D%22%24nodes%22%2F%3E%20%3C%2Fxsl%3Aotherwise%3E%20%3C%2Fxsl%3Achoose%3E%20%3C%2Fxsl%3Acopy%3E%20%3C%2Fxsl%3Afor-each%3E%20%3C%2Fxsl%3Awhen%3E%20%3Cxsl%3Aotherwise%3E%20%3Cxsl%3Aapply-templates%20select%3D%22%40%2A%7Cnode%28%29%22%20mode%3D%22screen-table%22%2F%3E%20%3C%2Fxsl%3Aotherwise%3E%20%3C%2Fxsl%3Achoose%3E%20%3C%2Fxsl%3Acopy%3E%20%3C%2Fxsl%3Atemplate%3E%20%3Cxsl%3Atemplate%20match%3D%22html%3Atd%5Bcontains%28concat%28%27%20%27%2C%20normalize-space%28%40class%29%2C%20%27%20%27%29%2C%20%27%20floating-table-cell%20%27%29%5D%22%20mode%3D%22screen-table%22%3E%20%3Cxsl%3Acopy%3E%20%3Cxsl%3Aapply-templates%20select%3D%22%40%2A%5Bname%28%29%20%21%3D%20%27class%27%5D%22%20mode%3D%22screen-table%22%2F%3E%20%3Cxsl%3Avariable%20name%3D%22full-class%22%20select%3D%22concat%28%27%20%27%2C%20normalize-space%28%40class%29%2C%20%27%20%27%29%22%2F%3E%20%3Cxsl%3Aattribute%20name%3D%22class%22%3E%20%3Cxsl%3Avalue-of%20select%3D%22normalize-space%28concat%28substring-before%28%24full-class%2C%20%27%20floating-table-cell%20%27%29%2C%20%27%20%27%2C%20substring-after%28%24full-class%2C%20%27%20floating-table-cell%20%27%29%29%29%22%2F%3E%20%3C%2Fxsl%3Aattribute%3E%20%3C%2Fxsl%3Acopy%3E%20%3C%2Fxsl%3Atemplate%3E%3C%2Fxsl%3Astylesheet%3E
%3Cxsl%3Astylesheet%20version%3D%221.0%22%20%20%20%20xmlns%3Axsl%3D%22http%3A%2F%2Fwww.w3.org%2F1999%2FXSL%2FTransform%22%20%20%20%20xmlns%3Ahtml%3D%22http%3A%2F%2Fwww.w3.org%2F1999%2Fxhtml%22%20%20%20%20xmlns%3D%22http%3A%2F%2Fwww.w3.org%2F1999%2Fxhtml%22%20%20%20%20id%3D%22ScrollableTable%22%20%20%20%20%3E%20%3Cxsl%3Apreserve-space%20elements%3D%22%2A%22%2F%3E%20%3Cxsl%3Atemplate%20match%3D%22%40%2A%7Cnode%28%29%22%3E%20%3Cxsl%3Acopy%3E%20%3Cxsl%3Aapply-templates%20select%3D%22%40%2A%7Cnode%28%29%22%2F%3E%20%3C%2Fxsl%3Acopy%3E%20%3C%2Fxsl%3Atemplate%3E%20%3Cxsl%3Atemplate%20match%3D%22html%3Atable%5Bcontains%28concat%28%27%20%27%2C%20normalize-space%28%40class%29%2C%20%27%20%27%29%2C%20%27%20scrollable-table%20%27%29%5D%22%3E%20%3Cxsl%3Avariable%20name%3D%22full-class%22%20select%3D%22concat%28%27%20%27%2C%20normalize-space%28%40class%29%2C%20%27%20%27%29%22%2F%3E%20%3Cxsl%3Avariable%20name%3D%22reduced-class%22%20select%3D%22normalize-space%28concat%28substring-before%28%24full-class%2C%20%27%20scrollable-table%20%27%29%2C%20%27%20%27%2C%20substring-after%28%24full-class%2C%20%27%20scrollable-table%20%27%29%29%29%22%2F%3E%20%3Cdiv%3E%20%3Cxsl%3Aapply-templates%20select%3D%22%40%2A%5Bname%28%29%20%21%3D%20%27class%27%5D%22%2F%3E%20%3Cxsl%3Aattribute%20name%3D%22class%22%3E%20%3Cxsl%3Avalue-of%20select%3D%22normalize-space%28concat%28%24reduced-class%2C%20%27%20scrollable-table-header%20scrollable-table-header-enabled%27%29%29%22%2F%3E%20%3C%2Fxsl%3Aattribute%3E%20%3Cp%20class%3D%22scrollable-toggle%22%3E%20%3Clabel%3E%20%3Cinput%20type%3D%22checkbox%22%20checked%3D%22checked%22%2F%3E%20Auto-fit%20to%20window%20%20%20%20%20%20%20%20%20%20%20%20%20%20%20%20%3C%2Flabel%3E%20%3C%2Fp%3E%20%3Ctable%20class%3D%22%7B%24reduced-class%7D%22%3E%20%3Cxsl%3Aapply-templates%20select%3D%22html%3Acol%7Chtml%3Acolgroup%7Chtml%3Athead%22%2F%3E%20%3C%2Ftable%3E%20%3C%2Fdiv%3E%20%3Cdiv%3E%20%3Cxsl%3Aapply-templates%20select%3D%22%40%2A%5Bname%28%29%20%21%3D%20%27class%27%5D%22%2F%3E%20%3Cxsl%3Aattribute%20name%3D%22class%22%3E%20%3Cxsl%3Avalue-of%20select%3D%22normalize-space%28concat%28%24reduced-class%2C%20%27%20scrollable-table-container%20scrollable-table-container-enabled%27%29%29%22%2F%3E%20%3C%2Fxsl%3Aattribute%3E%20%3Ctable%20class%3D%22%7B%24reduced-class%7D%22%3E%20%3Cxsl%3Aapply-templates%20select%3D%22node%28%29%22%2F%3E%20%3C%2Ftable%3E%20%3C%2Fdiv%3E%20%3C%2Fxsl%3Atemplate%3E%3C%2Fxsl%3Astylesheet%3E
%3Cxsl%3Astylesheet%20version%3D%221.0%22%20%20%20%20xmlns%3Axsl%3D%22http%3A%2F%2Fwww.w3.org%2F1999%2FXSL%2FTransform%22%20%20%20%20xmlns%3Ahtml%3D%22http%3A%2F%2Fwww.w3.org%2F1999%2Fxhtml%22%20%20%20%20xmlns%3D%22http%3A%2F%2Fwww.w3.org%2F1999%2Fxhtml%22%20%20%20%20id%3D%22ToggledContent.Capture%22%20%20%20%20%3E%20%3Cxsl%3Apreserve-space%20elements%3D%22%2A%22%2F%3E%20%3Cxsl%3Atemplate%20match%3D%22%40%2A%7Cnode%28%29%22%3E%20%3Cxsl%3Acopy%3E%20%3Cxsl%3Aapply-templates%20select%3D%22%40%2A%7Cnode%28%29%22%2F%3E%20%3C%2Fxsl%3Acopy%3E%20%3C%2Fxsl%3Atemplate%3E%20%3Cxsl%3Akey%20%20%20%20%20%20%20%20%20name%3D%22controls%22%20%20%20%20%20%20%20%20%20match%3D%22%2A%5B%28local-name%28%29%3D%27a%27%20or%20local-name%28%29%3D%27span%27%29%20and%20%40tc-control%20and%20contains%28concat%28%27%20%27%2C%20normalize-space%28%40class%29%2C%20%27%20%27%29%2C%20%27%20tc-static%20%27%29%20and%20not%28%40href%29%20and%20%28%40tc-toggled%3D0%20or%20%40tc-toggled%3D1%29%5D%22%20%20%20%20%20%20%20%20%20use%3D%22%40tc-control%22%20%20%20%20%20%20%20%20%2F%3E%20%3Cxsl%3Atemplate%20match%3D%22%2A%5Bkey%28%27controls%27%2C%20%40tc-control%29%5D%22%3E%20%3Cxsl%3Acopy%3E%20%3Cxsl%3Aapply-templates%20select%3D%22%40%2A%22%2F%3E%20%3Cimg%20class%3D%22symbol%22%20title%3D%22%22%3E%20%3Cxsl%3Acopy-of%20select%3D%22.%2Fancestor-or-self%3A%3A%2A%2F%40xml%3Abase%22%2F%3E%20%3Cxsl%3Achoose%3E%20%3Cxsl%3Awhen%20test%3D%22%40tc-toggled%20%3D%200%22%3E%20%3Cxsl%3Aattribute%20name%3D%22src%22%3E..%2Fimages%2Fnode-closed.gif%3C%2Fxsl%3Aattribute%3E%20%3Cxsl%3Aattribute%20name%3D%22alt%22%3E%2B%3C%2Fxsl%3Aattribute%3E%20%3C%2Fxsl%3Awhen%3E%20%3Cxsl%3Awhen%20test%3D%22%40tc-toggled%20%3D%201%22%3E%20%3Cxsl%3Aattribute%20name%3D%22src%22%3E..%2Fimages%2Fnode-opened.gif%3C%2Fxsl%3Aattribute%3E%20%3Cxsl%3Aattribute%20name%3D%22alt%22%3E-%3C%2Fxsl%3Aattribute%3E%20%3C%2Fxsl%3Awhen%3E%20%3C%2Fxsl%3Achoose%3E%20%3C%2Fimg%3E%20%3Cxsl%3Aapply-templates%20select%3D%22node%28%29%22%2F%3E%20%3C%2Fxsl%3Acopy%3E%20%3C%2Fxsl%3Atemplate%3E%20%3Cxsl%3Atemplate%20match%3D%22%2A%5B%40tc-content%5D%22%3E%20%3Cxsl%3Avariable%20name%3D%22toggles%22%20select%3D%22key%28%27controls%27%2C%20%40tc-content%29%22%2F%3E%20%3Cxsl%3Achoose%3E%20%3Cxsl%3Awhen%20test%3D%22count%28%24toggles%29%3D0%22%3E%20%3Cxsl%3Acopy%3E%3Cxsl%3Aapply-templates%20select%3D%22%40%2A%7Cnode%28%29%22%2F%3E%3C%2Fxsl%3Acopy%3E%20%3C%2Fxsl%3Awhen%3E%20%3Cxsl%3Aotherwise%3E%20%3Cxsl%3Avariable%20name%3D%22togglestate%22%20select%3D%22number%28%24toggles%5B1%5D%2F%40tc-toggled%29%22%2F%3E%20%3Cxsl%3Avariable%20name%3D%22full-class%22%20select%3D%22concat%28%27%20%27%2C%20normalize-space%28%40class%29%2C%20%27%20%27%29%22%2F%3E%20%3Cxsl%3Acopy%3E%20%3Cxsl%3Aapply-templates%20select%3D%22%40%2A%5Bname%28%29%20%21%3D%20%27class%27%5D%22%2F%3E%20%3Cxsl%3Aattribute%20name%3D%22class%22%3E%20%3Cxsl%3Achoose%3E%20%3Cxsl%3Awhen%20test%3D%22%24togglestate%20%3D%201%22%3E%20%3Cxsl%3Avalue-of%20select%3D%22normalize-space%28%24full-class%29%22%2F%3E%20%3C%2Fxsl%3Awhen%3E%20%3Cxsl%3Awhen%20test%3D%22%24togglestate%20%3D%200%22%3E%20%3Cxsl%3Avalue-of%20select%3D%22normalize-space%28concat%28%24full-class%2C%20%27%20tc-invisible%27%29%29%22%2F%3E%20%3C%2Fxsl%3Awhen%3E%20%3Cxsl%3Aotherwise%3E%20%3Cxsl%3Avalue-of%20select%3D%22normalize-space%28%24full-class%29%22%2F%3E%20%3C%2Fxsl%3Aotherwise%3E%20%3C%2Fxsl%3Achoose%3E%20%3C%2Fxsl%3Aattribute%3E%20%3Cxsl%3Aapply-templates%20select%3D%22node%28%29%22%2F%3E%20%3C%2Fxsl%3Acopy%3E%20%3C%2Fxsl%3Aotherwise%3E%20%3C%2Fxsl%3Achoose%3E%20%3C%2Fxsl%3Atemplate%3E%20%3Cxsl%3Atemplate%20match%3D%22html%3Aform%5B%40tc-control%20and%20not%28contains%28concat%28%27%20%27%2C%20%40class%2C%20%27%20%27%29%2C%20%27%20selectbox-manipulator%20%27%29%29%5D%22%3E%20%3Cform%20onsubmit%3D%22return%20ToggledContent.Capture.handle\_onsubmit%28this%29%3B%22%20tc-captured%3D%221%22%3E%20%3Cxsl%3Aapply-templates%20select%3D%22%40%2A%7Cnode%28%29%22%2F%3E%20%3C%2Fform%3E%20%3C%2Fxsl%3Atemplate%3E%20%3Cxsl%3Akey%20%20%20%20%20%20%20%20%20name%3D%22grayed-out-inputs%22%20%20%20%20%20%20%20%20%20match%3D%22html%3Ainput%5B%40type%20%21%3D%20%27hidden%27%20and%20%40disabled%20and%20ancestor%3A%3A%2A%5B%40tc-content%20or%20%40tc-depend%20or%20%40tc-control%5D%5D%22%20%20%20%20%20%20%20%20use%3D%22generate-id%28%29%22%20%20%20%20%20%20%20%20%2F%3E%20%3Cxsl%3Akey%20%20%20%20%20%20%20%20%20name%3D%22grayed-out-selects%22%20%20%20%20%20%20%20%20%20match%3D%22html%3Aselect%5B%40disabled%20and%20ancestor%3A%3A%2A%5B%40tc-content%20or%20%40tc-depend%20or%20%40tc-control%5D%5D%22%20%20%20%20%20%20%20%20use%3D%22generate-id%28%29%22%20%20%20%20%20%20%20%20%2F%3E%20%3Cxsl%3Atemplate%20match%3D%22html%3Ainput%5Bkey%28%27grayed-out-inputs%27%2C%20generate-id%28%29%29%5D%22%3E%20%3Cinput%20tc-default-disabled%3D%221%22%3E%20%3Cxsl%3Aapply-templates%20select%3D%22%40%2A%7Cnode%28%29%22%2F%3E%20%3C%2Finput%3E%20%3C%2Fxsl%3Atemplate%3E%20%3Cxsl%3Atemplate%20match%3D%22html%3Aselect%5Bkey%28%27grayed-out-selects%27%2C%20generate-id%28%29%29%5D%22%3E%20%3Cselect%20tc-default-disabled%3D%221%22%3E%20%3Cxsl%3Aapply-templates%20select%3D%22%40%2A%7Cnode%28%29%22%2F%3E%20%3C%2Fselect%3E%20%3C%2Fxsl%3Atemplate%3E%3C%2Fxsl%3Astylesheet%3E
